# Lower frequency of TLR9 variant associated with protection from breast cancer among African Americans

**DOI:** 10.1371/journal.pone.0183832

**Published:** 2017-09-08

**Authors:** Madison R. Chandler, Kimberly S. Keene, Johanna M. Tuomela, Andres Forero-Torres, Renee Desmond, Katri S. Vuopala, Kevin W. Harris, Nancy D. Merner, Katri S. Selander

**Affiliations:** 1 Harrison School of Pharmacy, Auburn University, Auburn, AL, United States of America; 2 Department of Radiation Oncology, University of Alabama at Birmingham, Birmingham, AL, United States of America; 3 Department of Cell Biology and Anatomy, Institute of Biomedicine, University of Turku, Turku, Finland; 4 Department of Medicine, Division of Hematology & Oncology, University of Alabama at Birmingham, Birmingham, AL, United States of America; 5 Comprehensive Cancer Center, University of Alabama at Birmingham, Birmingham, AL, United States of America; 6 Department of Medicine, Division of Preventive Medicine, University of Alabama at Birmingham, Birmingham, AL, United States of America; 7 Department of Pathology, Lapland Central Hospital, Rovaniemi, Finland; 8 Birmingham Veterans Affairs Medical Center, Birmingham, AL, United States of America; 9 Department of Pathobiology, College of Veterinary Medicine, Auburn University, Auburn, AL, United States of America; 10 Department of Chemistry, University of Alabama at Birmingham, Birmingham, AL, United States of America; University of South Alabama Mitchell Cancer Institute, UNITED STATES

## Abstract

**Introduction:**

Toll-like receptor 9 (TLR9) is an innate immune system DNA-receptor that regulates tumor invasion and immunity *in vitro*. Low tumor TLR9 expression has been associated with poor survival in Caucasian patients with triple negative breast cancer (TNBC). African American (AA) patients with TNBC have worse prognosis than Caucasians but whether this is due to differences in tumor biology remains controversial. We studied the prognostic significance of tumor Toll like receptor-9 (TLR9) protein expression among African American (AA) triple negative breast cancer (TNBC) patients. Germline *TLR9* variants in European Americans (EAs) and AAs were investigated, to determine their contribution to AA breast cancer risk.

**Methods:**

TLR9 expression was studied with immunohistochemistry in archival tumors. Exome Variant Server and The Cancer Genome Atlas were used to determine the genetic variation in the general EA and AA populations, and AA breast cancer cases. Minor allele frequencies (MAFs) were compared between EAs (n = 4300), AAs (n = 2203), and/or AA breast cancer cases (n = 131).

**Results:**

Thirty-two *TLR9* variants had a statistically significant MAF difference between general EAs and AAs. Twenty-one of them affect a CpG site. Rs352140, a variant previously associated with protection from breast cancer, is more common in EAs than AAs (p = 2.20E-16). EAs had more synonymous alleles, while AAs had more rare coding alleles. Similar analyses comparing AA breast cancer cases with AA controls did not reveal any variant class differences; however, three previously unreported *TLR9* variants were associated with late onset breast cancer. Although not statistically significant, rs352140 was observed less frequently in AA cases compared to controls. Tumor TLR9 protein expression was not associated with prognosis.

**Conclusions:**

Tumor TLR9 expression is not associated with prognosis in AA TNBC. Significant differences were detected in *TLR9* variant MAFs between EAs and AAs. They may affect TLR9 expression and function. Rs352140, which may protect from breast cancer, is 1.6 X more common among EAs. These findings call for a detailed analysis of the contribution of TLR9 to breast cancer pathophysiology and health disparities.

## Introduction

Toll Like Receptor-9 (TLR9) is an endosomal DNA receptor that belongs to the innate immune system. It recognizes and reacts to both microbial and vertebrate (self) DNA that has entered cells, either during microbial infections or during cell death.[1–3] Activation of TLR9 by DNA stimulation results in a rapid and a robust inflammatory reaction, with increased production of Th1-biased inflammatory mediators that activate the adaptive immune system.[4–6] The outcome of this reaction is an immunological elimination of the invading microbe and the infected cells.[4, 7] A similar inflammatory response occurs during sterile tissue damage, such as infarction or trauma.[8, 9]

TLR9 is also widely expressed in various human cancer cell lines and clinical tumors, including breast, brain, lung, ovarian, prostate, kidney and GI-tract cancers.[10–19] Stimulation of TLR9 with synthetic DNA-ligands (CpG-oligonucleotides) or cell-derived DNA has been shown to induce cytokine expression in TLR9-expressing cancer cells *in vitro*.[20, 21] In addition, both synthetic TLR9-ligands as well as cell-derived DNA induce cancer invasion *in vitro*.[3, 10, 12, 22, 23] TLR9 can also regulate cancer cell invasion ligand-independently.[15, 24] The clinical relevance of these findings and the significance of TLR9 in cancer pathophysiology has however, remained unclear. A recent meta-analysis by Wan *et al*. suggested that certain *TLR9* variants (such as rs187084) might be associated with an elevated cancer risk, especially in cervical cancer.[25] Other *TLR9* variants (such as rs352140 and rs5743836, respectively) were suggested to be protective of breast and digestive system cancers.[25] Currently, quite little is known about the functional effects of the various *TLR9* genetic variants on cancer or other cells.[26–29] When studied with immunohistochemistry, tumor TLR9 expression can indicate cancer-specific prognoses; on one hand, high TLR9 expression was associated with decreased survival in brain, prostate and esophageal cancers.[11, 18, 30] On the other hand, in triple negative breast cancer (TNBC), renal cell carcinoma, mucoepidermoid salivary gland carcinoma, and most recently in pancreatic cancer, *low* tumor TLR9 protein expression is associated with poor survival.[13, 15, 31–33]. This suggests that high tumor TLR9 expression *protects* from relapses in these malignancies. The mechanisms for such protection are unclear.

Of all breast cancer patients, those with TNBC have the worst prognosis.[34–36] These tumors are generally aggressive and lack the expression of drug targets, such as estrogen receptor (ER), progesterone receptor (PR) and human epidermal growth factor receptor-2 (HER2).[34–36] TNBC is especially troubling in African American (AA) women. Firstly, large population-based studies have identified a higher proportion of TNBC among premenopausal AA women.[37] Secondly, AA women have higher mortality rate from breast cancer than Caucasian or European Americans (EA) even when socioeconomic factors are taken into account. [37] Although the results of a recent study suggested that TNBC in AA women is not a unique disease compared to TNBC in Caucasian women, biological differences have been indeed detected.[37–39] TLR9 has not been studied in breast or other cancer disparities previously. The aim of this study was to evaluate tumor TLR9 expression among AA TNBC patients and to assess its relationship to survival and recurrence in this patient population. We also aimed to identify the landscape of germline *TLR9* variants in both EAs and AAs, and determine if such variants contribute to AA breast cancer risk.

## Materials and methods

### Breast cancer specimens

AA females with TNBC treated between 2000 and 2008 at The University of Alabama at Birmingham (UAB; Birmingham, Alabama, U.S.A) and with tissue available for analysis were identified in a pathology database. The working data was for 51 patients, but the follow-up data was available only for 43–49 subjects. The associated paraffin-embedded tumor blocks were processed for immunohistochemical staining and evaluation of TLR9 expression using standard techniques and as previously explained.[15, 40] Specifically, we used the following definition of TNBC to select the tumors for the immunohistochemical stainings: Tumors exhibiting any *nuclear* estrogen/progesterone receptor expression in invasive tumor cells were considered as steroid receptor-positive and excluded. Membranous HER2 expression was also studied by means of immunohistochemistry (IHC) and if a specimen exhibited a HER2-positive result in IHC, the HER2 gene amplification status was determined by means of chromogenic *in situ* hybridization. HER2-positive tumors were excluded. All patients got standard treatment and care, consisting of surgery, radiation and chemotherapy (adjuvant or neo-adjuvant). The research was approved by the UAB Institutional Review Board and by the Ethics Council of The Northern Ostrobothnia Hospital District (Oulu, Finland).

### Tumor TLR9 staining and scoring

Immunohistochemical staining for TLR9 and scoring of the staining intensities were performed as previously described.[40] Briefly, tissue sections of 5 μm in thickness were cut from the formalin-fixed, archived, paraffin-embedded tissue blocks. Immunohistochemical staining was performed with a LabVision Autostainer™ (LabVision, Fremont, CA, USA), using the Envision™ Detection System (K500711; Dako Denmark A/S, Glostrup, Denmark). The antibody used was the anti-TLR9 (Img-305A, diluted 1:200, Imgenex, San Diego, CA, U.S.A). TLR9-staining intensity scores (0–16) were divided into low (<8) and high (≥8), according to the previously used criteria.[15, 25, 40] Clinical information was obtained from patient records. TLR9 expression scores were compared with retrospective outcome data, including ipsilateral breast cancer, disease-free and overall survival. TLR9 scores and the associated survival data are shown in S1 Table.

### Statistical analysis of TLR9 protein expression data

Clinical and pathologic characteristic were summarized as means (sd) for continuous variables and frequencies for categorical variables. The relationship between TLR9 and survival, disease-free survival and ipsilateral breast tumor recurrence were evaluated using the log-rank test and Kaplan Meier survival curves with TLR9 dichotomized at the approximate median value.

### Genetic analyses

Firstly, *TLR9* variants reported in the Exome Variant Server (EVS; http://evs.gs.washington.edu/EVS/; release ESP6500SI-V2) of the National Heart Lung and Blood Institute (NHLBI) GO Exome Sequencing Project (ESP) (https://esp.gs.washington.edu/drupal/) were recorded to determine variant calls and frequencies in both AAs and EAs, which represented the general population for each ethnicity. Overall, all called variants were compared between the two ethnicities to identify *variants with statistically significant different allele frequencies*. The statistical analyses involved Fisher Exact tests using the program R (R 3.3.2 GUI 1.68 Mavericks build (7288)). To determine if TLR9 variants play a role in AA breast cancer susceptibility, a request (*#44682–1*) for The Cancer Genome Atlas (TCGA) data access was submitted and project (*#10805)* was approved. Sequencing data from select breast cancer-affected individuals was collected from the Genomic Data Commons (GDC) Data Portal.[41, 42] Cases were filtered for ‘Project:’ TCGA-BRCA, ‘Disease Type:’ Breast Invasive Carcinoma, ‘Race:’ black or AA, and ‘Samples Sample Type:’ Blood Derived Normal. Files were filtered for ‘Experimental Strategy:’ WXS (whole exome sequencing), and ‘Data Format:’ BAM (binary sequence alignment-mapping). BAM files were produced by mapping/aligning sequencing data to the GRCh38.p0 reference genome using Burrows-Wheeler Aligner with Mark Duplicates and Cocleaning.[41] After filtering, 170 whole-exome BAM files remained. The manifest of the selected BAM files was downloaded from the GDC Data Portal; 131 of the 170 whole-exome BAM files were downloaded using the GDC Data Transfer Tool (version 1.2.0) [43]. HaplotypeCaller from the Genome Analysis Toolkit (GATK; version 3.6) was used to call variants from the downloaded BAM files and to generate Variant Calling Format (VCF) files for the whole-exome [44]. Variants located on chromosome 3 between base pair 52221079 and 52226163 were extracted from the whole-exome VCF files using VCFTools (version 0.1.12a).[45] The extracted VCF files that contain variants located in *TLR9* were compressed and indexed using Tabix (version 0.2.6).[46] Ultimately, extracted VCF files were merged for all 131 AA breast cancer cases and allele frequencies were calculated and compared to the frequencies reported in AAs in EVS. Clinical data for the breast cancer-affected individuals was collected from the GDC Data Portal (S2 Table).[41]

## Results

### No association between tumor TLR9 expression status and survival among AA TNBC patients

**Fig 1:**
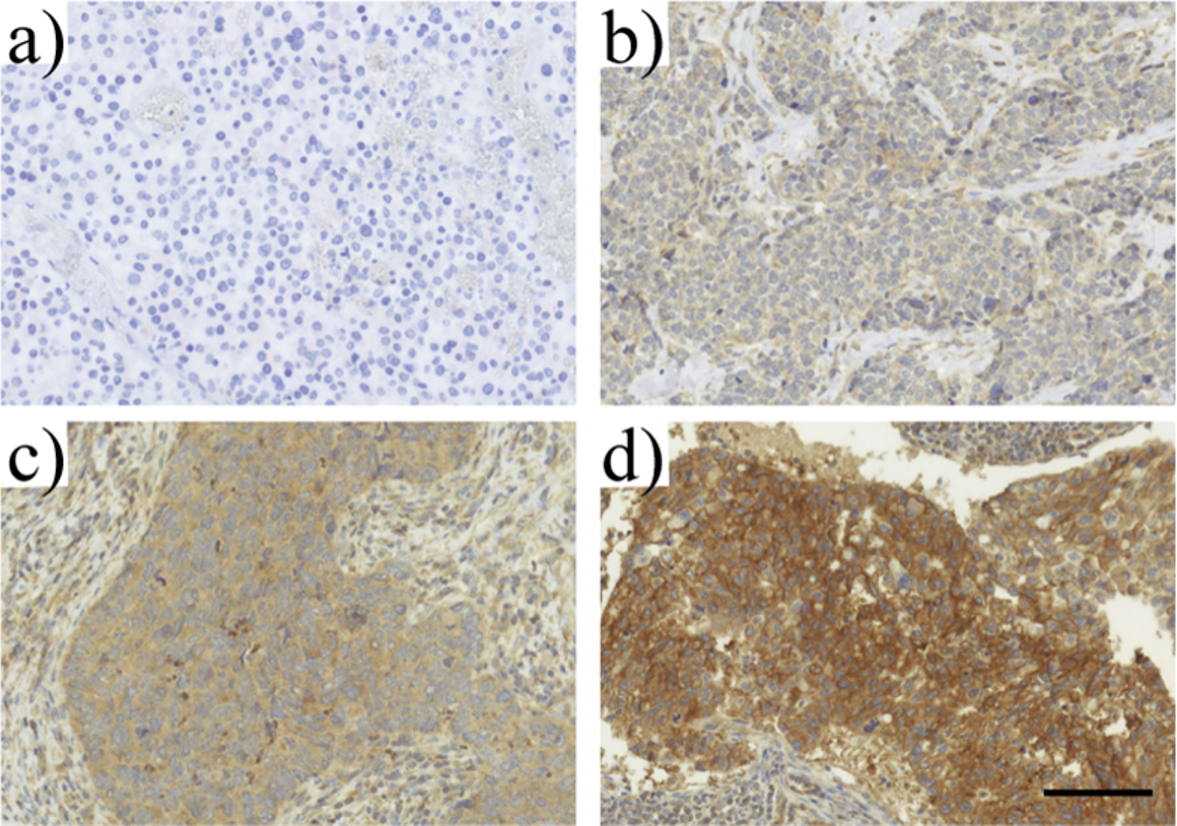
Examples of immunohistochemical TLR9 stainings in tissue sections of paraffin-embedded TNBC blocks from African American women. The staining examples represent a) negative, b) low, c) intermediate and d) high TLR9 expressing TNBC tumors. Staining results in a & b represent the low TLR9-group (TLR9 score ≤ 8), whereas c & d represent the high tumor TLR9-group (TLR9 score > 8).

**Fig 2:**
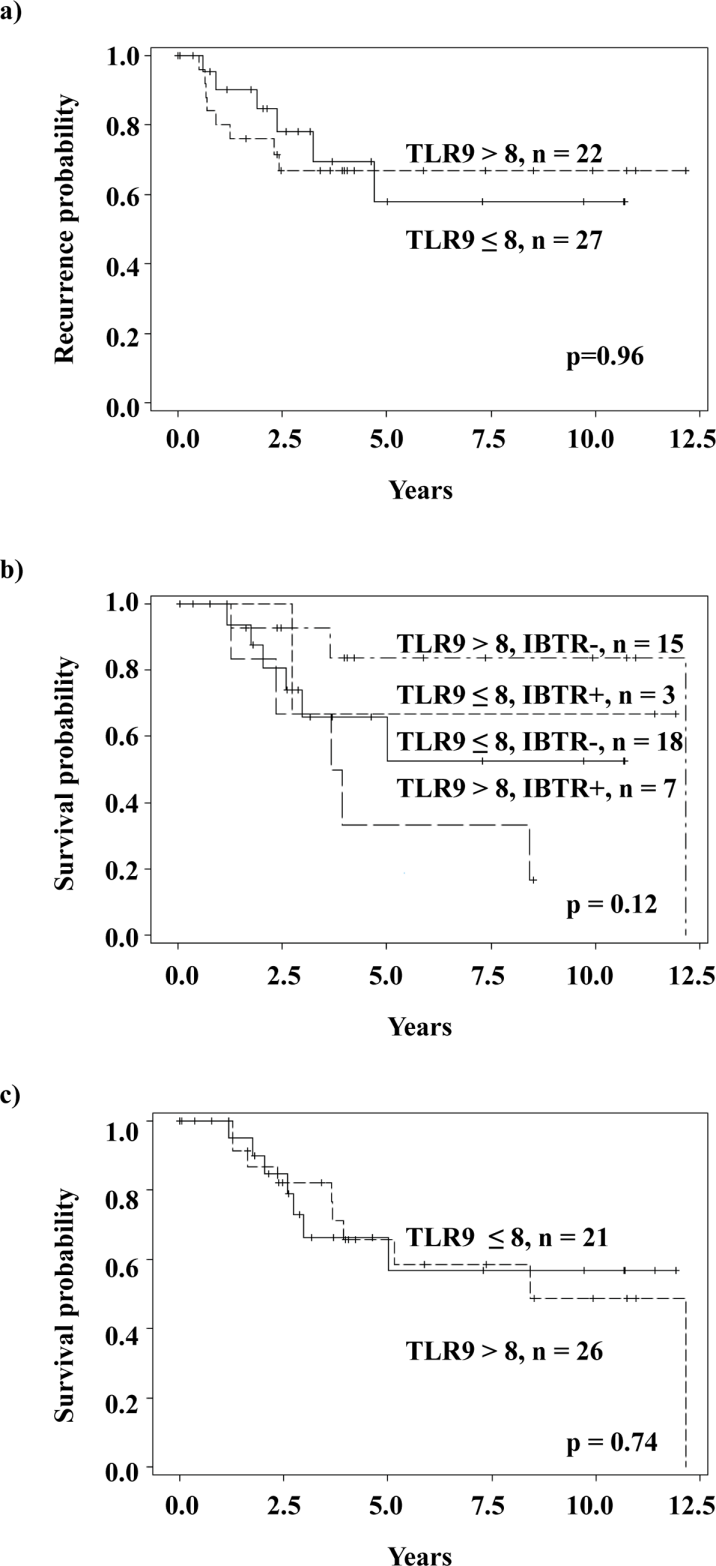
Patient outcomes stratified by tumor TLR9 expression status. a) Breast cancer recurrence probability stratified by median tumor TLR9 expression status, b) survival probability stratified by ipsilateral breast cancer (IBTR) and tumor TLR9 expression status and c) breast cancer specific-survival probability stratified by tumor TLR9 expression status (n = 43).

The baseline and clinico-pathologic characteristics of the studied breast cancer patient population are shown in S3 Table. Fifty-one patients had tissue evaluable for review. Median age at the time of diagnosis was 51 years. Most patients (n = 31, 72%) were early stage (I–II), and the majority of the patients were also node-negative (n = 30, 67%). Most patients had ductal infiltrating carcinoma (n = 43, 90%). All patients received standard of care, consisting of surgery, radiation and chemotherapy. Most patients had mastectomy (n = 28, 61%), with the minority undergoing lumpectomy. All patients received some form of chemotherapy (26% in the neo-adjuvant setting, 74% adjuvant systemic therapy). Radiation therapy was used in 66% of patients in the post-operative setting. At a median follow-up of 3.5 years, 25 patients (58%) experienced some type of recurrence. Eleven patients (25%) had ipsilateral breast tumor recurrence. TLR9 staining in the paraffin-embedded tumor sections was performed as previously described.[40] Examples of high and low TLR9 staining patterns in the studied AA TNBC specimens are shown in Fig 1. The TLR9 staining range was similar as previously detected in the EA cohort (data not shown). However, unlike previously shown for Caucasian TNBC patients,[15] tumor TLR9 protein expression score was not significantly associated with breast cancer recurrence, ipsilateral breast cancer or breast cancer-specific survival, Fig 2.

### *TLR9* variant differences in general AA and EA populations

A total of 147 different variants were called in EVS between both ethnicities; this included 31 variants that were detected in both EAs and AAs, as well as 65 variants that were unique to EAs and 51 variants that were unique to AAs. Of the 31 overlapping variants, 15 had no statistically significant difference in the minor allele frequencies (MAFs) between ethnicities; nine variants had statistically significant higher MAFs in AAs compared to EAs (Table 1), and six variants had statistically significant higher MAFs in EAs (Table 2). Of the 65 variants that were unique to EAs, 62 were extremely rare with MAFs between 0.01 and 0.06% with no significant MAF difference between ethnicities, and three variants had a statistically significant higher MAF compared to AAs (Table 2). Regarding the 51 unique AA variants, 37 were extremely rare with MAFs between 0.02 and 0.04% with no significant MAF difference between ethnicities, and 14 variants had a statistically significant higher MAFs compared to EAs (Table 1).

**Table 1:** Variants with a statistically significant MAF in AAs.

Variant Status	GRCh38 Position	rs ID	Alleles	GVS Function	cDNA Change	Protein Change	PolyPhen2 (Class:Score)	Clinical Link	CpG site	EVS MAF (%)		p values	Odds Ratio
										EA	AA		
AA and EA overlapping variants	3:52224303	rs5743842	G>A	missense	c.13C>T	p.(R5C)	benign:0.002	unknown	disrupt	0.02	3.79	2.20E-16	0.01 CI_95_[0.0–0.0]
	3:52223812	rs72959313	G>A	coding-synonymous	c.504C>T	p.(A168 =)	unknown	unknown	no effect	0.01	1.29	2.20E-16	0.01 CI_95_[0.0–0.1]
	3:52223791	rs138035523	G>A	coding-synonymous	c.525C>T	p.(D175 =)	unknown	unknown	disrupt	0.01	0.34	9.56E-07	0.03 CI_95_[0.0–0.2]
	3:52223620	rs148139239	G>A	coding-synonymous	c.696C>T	p.(I232 =)	unknown	unknown	disrupt	0.05	0.70	2.69E-11	0.07 CI_95_[0.0–0.2]
	3:52223167	rs35654187	C>T	coding-synonymous	c.1149G>A	p.(T383 =)	unknown	unknown	disrupt	0.40	1.02	3.57E-05	0.38 CI_95_[0.2–0.6]
	3:52221731	rs148303873	C>T	missense	c.2585G>A	p.(G862E)	benign:0.414	unknown	no effect	0.01	0.45	5.49E-09	0.03 CI_95_[0.0–0.2]
	3:52221728	rs5743845	C>T	missense	c.2588G>A	p.(R863Q)	benign:0.041	unknown	disrupt	0.42	3.52	2.20E-16	0.12 CI_95_[0.1–0.2]
	3:52221649	rs149908506	G>A	coding-synonymous	c.2667C>T	p.(N889 =)	unknown	unknown	disrupt	0.01	0.14	7.49E-03	0.09 CI_95_[0.0–0.7]
	3:52221431	rs201773280	C>T	missense	c.2885G>A	p.(R962H)	probably-damaging:1.0	unknown	disrupt	0.01	0.20	4.07E-04	0.06 CI_95_[0.0–0.4]
Unique AA variants	3:52224246	rs146965009	G>A	coding-synonymous	c.70C>T	p.(L24 =)	unknown	unknown	no effect	0	0.25	6.67E-06	Inf CI_95_[4.9-Inf]
	3:52224196	rs151147353	G>A	coding-synonymous	c.120C>T	p.(H40 =)	unknown	unknown	disrupt	0	0.09	1.32E-02	Inf CI_95_[1.3-Inf]
	3:52223875	rs116310431	G>T	coding-synonymous	c.441C>A	p.(S147 =)	unknown	unknown	no effect	0	2.34	2.20E-16	Inf CI_95_[54.7-Inf]
	3:52223704	rs150009336	C>G	coding-synonymous	c.612G>C	p.(L204 =)	unknown	unknown	no effect	0	0.07	3.89E-02	Inf CI_95_[0.8-Inf]
	3:52223649	rs201564821	C>T	missense	c.667G>A	p.(E223K)	benign:0.016	unknown	no effect	0	0.18	1.73E-04	Inf CI_95_[3.3-Inf]
	3:52223569	rs140856643	G>A	coding-synonymous	c.747C>T	p.(L249 =)	unknown	unknown	disrupt	0	0.32	2.59E-07	Inf CI_95_[6.5-Inf]
	3:52223223	rs373946909	C>T	missense	c.1093G>A	p.(A365T)	possibly-damaging:0.629	unknown	disrupt	0	0.07	3.89E-02	Inf CI_95_[0.8-Inf]
	3:52222605	rs115440379	C>T	missense	c.1711G>A	p.(V571M)	probably-damaging:0.992	unknown	disrupt	0	0.39	9.99E-09	Inf CI_95_[8.1-Inf0]
	3:52222431	rs34399053	C>T	missense	c.1885G>A	p.(G629S)	benign:0.022	unknown	disrupt	0	0.16	5.10E-04	Inf CI_95_[2.8-Inf]
	3:52222299	rs144698131	G>C	missense	c.2017C>G	p.(L673V)	probably-damaging:0.999	unknown	introduces	0	0.52	1.48E-11	Inf CI_95_[11.3-Inf]
	3:52222141	rs142377483	C>G	missense	c.2175G>C	p.(K725N)	benign:0.005	unknown	introduces	0	0.14	1.51E-03	Inf CI_95_[2.3-Inf]
	3:52221424	rs148465111	G>A	coding-synonymous	c.2892C>T	p.(D964 =)	unknown	unknown	disrupt	0	0.07	3.89E-02	Inf CI_95_[0.8-Inf]
	3:52221350	rs201478487	C>T	missense	c.2966G>A	p.(R989H)	benign:0.302	unknown	disrupt	0	0.20	5.85E-05	Inf CI_95_[3.9-Inf]
	3:52221292	rs200965458	C>G	missense	c.3024G>C	p.(Q1008H)	probably-damaging:0.987	unknown	no effect	0	0.20	5.85E-05	Inf CI_95_[3.9-Inf]

Accession #: NM_017442

**Table 2:** Variants with a statistically significant MAF in EAs.

Variant Status	GRCh38 Position	rs ID	Alleles	GVS Function	cDNA Change	Protein Change	PolyPhen2 (Class:Score)	Clinical Link	CpG site	EVS MAF (%)		p values	Odds Ratio
										EA	AA		
EA and AA overlapping variants	3:52224140	unknown	R>A1	frameshift	c.175del1	p.(A59Qfs*54)	unknown	unknown	no effect	0.67	0.33	1.52E-02	0.49 CI_95_[0.3–0.9]
	3:52222789	rs35342983	C>T	coding-synonymous	c.1527G>A	p.(S509 =)	unknown	unknown	disrupt	0.40	0.07	6.51E-04	5.69 CI_95_[1.8–29.0]
	3:52222681	rs352140	C>T	coding-synonymous	c.1635G>A	p.(P545 =)	unknown	unknown	disrupt	55.17*	34.52*	2.20E-16	2.33 CI_95_[2.2–2.5]
	3:52221802	rs138032346	G>A	coding-synonymous	c.2514C>T	p.(L838 =)	unknown	unknown	no effect	0.33	0.07	3.60E-03	4.79 CI_95_[1.5–24.7]
	3:52221376	rs445676	G>A	coding-synonymous	c.2940C>T	p.(Y980 =)	unknown	unknown	disrupt	1.73	0.41	2.20E-16	25.74 CI_95_[8.6–126.0]
	3:52221199	rs5743848	C>G	utr-3	c.*18G>C	NA	unknown	unknown	no effect	1.14	0.07	2.94E-14	16.9 CI_95_[5.6–83.4]
Unique EA variants	3:52225443	rs373979034	T>C	intron	c.3+84A>G	NA	unknown	unknown	no effect	0.57	0	1.56E-07	Inf CI_95_[6.1-Inf]
	3:52222519	rs143900156	C>T	coding-synonymous	c.1797G>A	p.(S599 =)	unknown	unknown	disrupt	0.16	0	3.85E-03	Inf CI_95_[1.7-Inf]
	3:52222270	rs143703479	G>A	coding-synonymous	c.2046C>T	p.(L682 =)	unknown	unknown	disrupt	0.10	0	3.33E-02	Inf CI_95_[1.0-Inf]

Accession #: NM_017442

* used T allele

Overall, EAs have a total of nine variants with a statistically significant higher MAF compared to AAs (Table 2). This included two noncoding and seven coding variants. The coding variants included six synonymous variants and a rare frameshift mutation. Interestingly, the frameshift mutation, p.(A59Qfs*54), results in a truncated protein of 111 amino acids, which is likely pathogenic, but currently not associated with any disease (Table 2). Furthermore, despite that the synonymous variants do not have an effect on the protein sequence, 83% (five out of the six) disrupt a CpG site (Table 2). Considering that CpG sites are known to be sites of DNA methylation that reduce gene expression when methylated, these variants could result in EA-specific TLR9 expression patterns.[47] Noteworthy, one of the synonymous variants is p.(P545 =), which is also known as rs352140 (Table 2); despite not being recognized as having a clinical link in EVS, rs352140 has been previously reported to have a protective effect against breast cancer.[25] Considering that rs352140 is significantly more common in EAs than AAs (Table 2), EAs likely benefit more from such protective effects than AAs. This variant disrupts a CpG site, thus its effect on expression could play a role in disease susceptibility.

AAs have a total of 23 variants with a statistically significant higher MAF compared to EAs, including 12 non-synonymous (missense) and 11 synonymous variants (Table 1). Seven of the missense variants are located in the extracellular domain, and five are in the cytoplasmic domain. Four are predicted to have strong pathogenic effects through Polyphen (Table 1) [48]; these are all extremely rare variants that could have detrimental effects on protein function. Of the 11 synonymous variants, 64% disrupt a CpG site. In fact, 70% of all the statistically significant AA variants affect a CpG site (Table 1). Overall, AAs had a significantly higher percentage of variants that had statistically significant higher MAFs compared to EAs (**P** = 6.43 X 10^−4^). Furthermore, there was a significant difference in the total number of allele counts for all variant types between ethnicities (Table 3). Overall, EAs had more synonymous alleles compared to AAs but AAs had significantly more rare coding alleles (Table 3).

**Table 3:** Comparison of allele counts between ethnicities for different variant classes.

Variant class	Cohort		Number of minor alleles reported	p value	Odds ratio
	Ethnicity	Size			
Total coding	EA	8600	5242	2.35E-05	1.13 CI_95_[1.1–1.2]
	AA	4406	2356		
Rare coding	EA	8600	348	1.01E-11	0.58 CI_95_[0.5–0.7]
	AA	4406	310		
Missense	EA	8600	95	2.20E-16	0.11 CI_95_[0.1–0.1]
	AA	4406	463		
Rare missense*	EA	8600	95	4.54E-16	0.34 CI_95_[0.3–0.4]
	AA	4406	143		
Probably damaging missense	EA	8600	16	2.20E-16	0.12 CI_95_[0.1–0.2]
	AA	4406	65		
Synonymous	EA	8600	5070	2.20E-16	1.38 CI_95_[1.3–1.5]
	AA	4406	1874		
Rare Synonymous*	EA	8600	176	1.41E-05	0.61 CI_95_[0.5–0.8]
	AA	4406	148		

*MAF less than or equal to 1%

### *TLR9* germline variants detected in TCGA AA breast cancer cases

A total of 21 different germline *TLR9* variants were detected in the 131 TCGA AA breast cancer cases (Table 4). This included eight missense and 13 synonymous variants. Only one of the missense variants (rs115440379) was predicted to be pathogenic, and it did not appear to be associated with breast cancer (Table 4 and S4 Table). Of the 13 synonymous variants, 8 disrupted a CpG site (Table 4); however, none appeared to be associated with breast cancer. Despite not being statistically significant, p.(P545 =) (rs352140) was observed even less frequently in the AA breast cancer cohort (Table 4 and S4 Table), which supports the previously reported protective effect.[25] Six of the breast cancer variants are common (MAF > 1%) in the general AA population with no statistical differences, and 15 are rare (MAF < 1%; Table 4 and S4 Table). Three of the latter were previously unreported and, individually, appeared to be associated with a later onset of breast cancer (diagnosed over the age of 45 years; Table 4 and S4 Table). Despite that aggregation analyses did not indicate a breast cancer association with particular *TLR9* variant classes, there were more rare coding variants reported in individuals diagnosed over the age of 45 compared to under 45 years of age (Table 5).

**Table 4:** TLR9 germline coding variants detected in 131 AA BC-affected individuals in the TCGA.

GRCh38 Position	rs ID	Alleles	GVS Function	cDNA Change	Protein Change	PolyPhen2 (Class:Score)	CpG site	MAF (%)				Comparing population controls (n = 2203) to TCGA BC cohorts						Comparing TCGA cohorts ≤ 45 years at diagnosis to > 45 years at diagnosis	
								TCGA BC cohort (n = 131)	TCGA BC cohort ≤ 45 years at diagnosis (n = 21)	TCGA BC cohort > 45 years at diagnosis (n = 110)	EVS AA—Population controls (n = 2203)	TCGA BC cohort (n = 131)		TCGA BC cohort ≤ 45 years at diagnosis (n = 21)		TCGA BC cohort > 45 years at diagnosis (n = 110)			
												p value	Odds ratio	p value	Odds ratio	p value	Odds ratio	p value	Odds ratio
3:52224303	rs5743842	G>A	missense	c.13C>T	p.(R5C)	benign:0.002	disrupt	2.67	4.76	2.27	3.79	0.499	0.70 CI_95_[0.3–1.5]	0.673	1.27 CI_95_[0.1–5.0]	0.357	0.59 CI_95_[0.2–1.4]	0.312	0.47 CI_95_[0.1–5.1]
3:52224246	rs146965009	G>A	coding-synonymous	c.70C>T	p.(L24 =)	unknown	no affect	0.76	0.00	0.91	0.25	0.163	3.07 CI_95_[0.3–14.2]	1.000	0.00 CI_95_[0.0–43.2]	0.125	3.66 CI_95_[0.4–16.9]	1.000	Inf CI_95_[0.0-Inf]
3:52223974	rs56116373	G>A	coding-synonymous	c.342C>T	p.(I114 =)	unknown	disrupt	0.38	0.00	0.45	0.05	0.159	8.42 CI_95_[0.1–162.6]	1.000	0.00 CI_95_[0.0–560.3]	0.136	10.04 CI_95_[0.2–193.4]	1.000	Inf CI_95_[0.0-Inf]
3:52223875	rs116310431	G>T	coding-synonymous	c.441C>A	p.(S147 =)	unknown	no affect	1.53	4.76	0.91	2.34	0.525	0.64 CI_95_[0.2–1.7]	0.261	2.09 CI_95_[0.2–8.2]	0.241	0.38 CI_95_[0.0–1.4]	0.1218	0.19 CI_95_[0.0–2.6]
3:52223812	rs72959313	G>A	coding-synonymous	c.504C>T	p.(A168 =)	unknown	no affect	1.53	0.00	1.82	1.29	0.776	1.18 CI_95_[0.3–3.2]	1.000	0.00 CI_95_[0.0–7.2]	0.535	1.41 CI_95_[0.4–3.9]	1.000	Inf CI_95_[0.1-Inf]
3:52223791	rs138035523	G>A	coding-synonymous	c.525C>T	p.(D175 =)	unknown	disrupt	0.76	0.00	0.91	0.34	0.247	2.25 CI_95_[0.2–9.8]	1.000	0.00 CI_95_[0.0–30.2]	0.192	2.68 CI_95_[0.3–11.7]	1.000	Inf CI_95_[0.0-Inf]
3:52223749	rs143323734	C>T	coding-synonymous	c.567G>A	p.(E189 =)	unknown	no affect	0.38	0.00	0.45	0.05	0.159	8.42 CI_95_[0.1–162.6]	1.000	0.00 CI_95_[0.0–560.3]	0.136	10.04 CI_95_[0.2–193.4]	1.000	Inf CI_95_[0.0-Inf]
3:52223619	rs137890561	C>T	missense	c.697G>A	p.(V233I)	benign:0.011	disrupt	0.38	0.00	0.45	0.05	0.159	8.42 CI_95_[0.1–162.6]	1.000	0.00 CI_95_[0.0–560.3]	0.136	10.04 CI_95_[0.2–193.4]	1.000	Inf CI_95_[0.0-Inf]
3:52223569	rs140856643	G>A	coding-synonymous	c.747C>T	p.(L249 =)	unknown	disrupt	0.38	0.00	0.45	0.32	0.517	0.37 CI_95_[0.0–2.2]	1.000	0.00 CI_95_[0.0–32.7]	0.519	1.43 CI_95_[0.0–9.5]	1.000	Inf CI_95_[0.0-Inf]
3:52223167	rs35654187	C>T	coding-synonymous	c.1149G>A	p.(T383 =)	unknown	disrupt	0.38	0.00	0.45	1.02	1.000	0.98 CI_95_[0.0–6.3]	1.000	0.00 CI_95_[0.0–9.2]	0.724	0.44 CI_95_[0.0–2.6]	1.000	Inf CI_95_[0.0-Inf]
3:52222681	rs352140	C>T	coding-synonymous	c.1635G>A	p.(P545 =)	unknown	disrupt	31.30*	33.33*	30.91*	34.52*	0.315	0.86 CI_95_[0.7–1.1]	1.000	0.95 CI_95_[0.5–1.9]	0.308	0.85 CI_95_[0.6–1.1]	0.8561	0.90 CI_95_[0.4–2.0]
3:52222605	rs115440379	C>T	missense	c.1711G>A	p.(V571M)	probably-damaging:0.992	disrupt	0.38	0.00	0.45	0.39	1.000	0.98 CI_95_[0.0–6.3]	1.000	0.00 CI_95_[0.0–26.3]	0.585	1.18 CI_95_[0.0–7.6]	1.000	Inf CI_95_[0.0-Inf]
3:52222206	rs145698725	G>A	missense	c.2110C>T	p.(R704W)	benign:0.058	disrupt	0.38	0.00	0.45	0.05	0.159	8.42 CI_95_[0.1–162.6]	1.000	0.00 CI_95_[0.0–560.3]	0.136	10.04 CI_95_[0.2–193.4]	1.000	Inf CI_95_[0.0-Inf]
3:52221826	rs372418469	C>G	coding-synonymous	c.2490G>C	p.(L830 =)	unknown	no affect	0.38	0.00	0.45	0.05	0.159	8.42 CI_95_[0.1–162.5]	1.000	0.00 CI_95_[0.0–560.1]	0.136	10.04 CI_95_[0.4–16.9]	1.000	Inf CI_95_[0.0-Inf]
3:52221731	rs148303873	C>T	missense	c.2585G>A	p.(G862E)	benign:0.414	no affect	0.76	0.00	0.91	0.45	0.353	1.69 CI_95_[0.2–7.0]	1.000	0.00 CI_95_[0.0–22.0]	0.282	2.01 CI_95_[0.2–8.4]	1.000	Inf CI_95_[0.0-Inf]
3:52221728	rs5743845	C>T	missense	c.2588G>A	p.(R863Q)	benign:0.041	disrupt	4.20	2.38	4.55	3.52	0.495	1.20 CI_95_[0.6–2.2]	1.000	0.67 CI_95_[0.0–4.0]	0.452	1.31 CI_95_[0.6–2.5]	1.000	1.95 CI_95_[0.3–86.7]
3:52221726	-	C>T	missense	c.2590G>A	p.(D864N)	benign:0.001	no affect	0.38	0.00	0.45	0.00	0.056	Inf CI_95_[0.4-Inf]	1.000	0.00 CI_95_[0.0-Inf]	0.048	Inf CI_95_[0.5-Inf]	1.000	Inf CI_95_[0.0-Inf]
3:52221723	-	C>T	missense	c.2593G>A	p.(E865K)	benign:0.116	no affect	0.38	0.00	0.45	0.00	0.056	Inf CI_95_[0.4-Inf]	1.000	0.00 CI_95_[0.0-Inf]	0.048	Inf CI_95_[0.5-Inf]	1.000	Inf CI_95_[0.0-Inf]
3:52221697	-	G>A	coding-synonymous	c.2619C>T	p.(F873 =)	unknown	disrupt	0.38	0.00	0.45	0.00	0.056	Inf CI_95_[0.4-Inf]	1.000	0.00 CI_95_[0.0-Inf]	0.048	Inf CI_95_[0.5-Inf]	1.000	Inf CI_95_[0.0-Inf]
3:52221649	rs149908506	G>A	coding-synonymous	c.2667C>T	p.(N889 =)	unknown	disrupt	0.38	0.00	0.45	0.14	0.333	2.83 CI_95_[0.1–23.3]	1.000	0.00 CI_95_[0.0–91.6]	0.2892	3.35 CI_95_[0.1–27.8]	1.000	Inf CI_95_[0.0-Inf]
3:52221376	rs445676	G>A	coding-synonymou	c.2940C>T	p.(Y980 =)	unknown	disrupt	0.38	0.00	0.45	0.41	1.000	0.93 CI_95_[0.0–5.9]	1.000	0.00 CI_95_[0.0–24.6]	0.6055	1.11 CI_95_[0.0–7.1]	1.000	Inf CI_95_[0.0-Inf]

Accession #: NM_017442

* used T allele

**Table 5:** Comparison of allele counts between AA cases and controls for different variant classes.

Variant class		Number of minor alleles reported		TCGA BC cohort > 45 years at diagnosis (n = 110)	EVS AA - Population controls (n = 2203)	Comparing population controls (n = 2203) to TCGA BC cohorts						Comparing TCGA cohorts ≤ 45 years at diagnosis to > 45 years at diagnosis	
		TCGA BC cohort (n = 131)	TCGA BC cohort ≤ 45 years at diagnosis (n = 21)			TCGA BC cohort (n = 131)		TCGA BC cohort ≤ 45 years at diagnosis (n = 21)		TCGA BC cohort > 45 years at diagnosis (n = 110)			
						p value	Odds ratio	p value	Odds ratio	p value	Odds ratio	p value	Odds ratio
Total	Coding	127	19	108	2356	0.411	0.90 CI_95_[0.7–1.1]	0.592	0.85 CI_95_[0.5–1.5]	0.514	0.92 CI_95_[0.7–1.2]	0.882	1.08 CI_95_[0.8-Inf]
	Missense	25	3	22	463	0.755	0.91 CI_95_[0.6–1.4]	0.796	0.64 CI_95_[0.0–2.9]	0.911	0.95 CI_95_[0.6–1.5]	0.777	1.40 CI_95_[0.4–7.6]
	Synonymous	102	16	86	1874	0.480	0.91 CI_95_[0.7–1.2]	0.775	0.90 CI_95_[0.5–1.6]	0.565	0.92 CI_95_[0.7–1.2]	1.000	1.02 CI_95_[0.5–2.1]
Rare*	Coding	18	0	18	310	1.000	0.98 CI_95_[0.6–1.6]	0.111	0.00 CI_95_[0.0–1.3]	0.505	1.16 CI_95_[0.7–1.9]	0.085	Inf CI_95_[0.8-Inf]
	Missense	7	0	7	143	0.856	0.82 CI_95_[0.3–1.8]	0.641	0.98 CI_95_[0.2–3.1]	1.000	0.98 CI_95_[0.4–2.1]	0.601	Inf CI_95_[0.5-Inf]
	Synonymous	11	0	11	148	0.482	1.25 CI_95_[0.6–2.3]	0.646	0.00 CI_95_[0.0–2.8]	0.191	1.49 CI_95_[0.7–2.8]	0.224	1.08 CI_95_[0.8-Inf]
Common**	Coding	109	19	90	2046	0.359	0.90 CI_95_[0.7–1.1]	1.000	0.97 CI_95_[0.5–1.7]	0.348	0.88 CI_95_[0.7–1.2]	0.760	0.90 CI_95_[0.5–1.7]
	Missense	18	3	15	320	0.903	0.95 CI_95_[0.5–1.6]	1.000	0.98 CI_95_[0.2–3.1]	1.000	0.94 CI_95_[0.5–1.6]	1.000	0.95 CI_95_[0.3–5.4]
	Synonymous	91	16	75	1726	0.361	0.89 CI_95_[0.7–1.1]	1.000	0.97 CI_95_[0.5–1.8]	0.320	0.87 CI_95_[0.7–1.1]	0.744	0.90 CI_95_[0.5–1.8]
Pathogenic	Missense	1	0	1	65	0.269	0.25 CI_95_[0.0–1.5]	1.000	0.00 CI_95_[0.0–6.4]	0.373	0.31 CI_95_[0.0–1.8]	1.000	Inf CI_95_[0.0-Inf]

*MAF less than or equal to 1%

**MAF greater than 1%

## Discussion

We described recently a novel, poor prognosis subtype in TNBC, as characterized by low tumor TLR9 expression upon diagnosis.[15] Specifically, patients whose TNBC tumors had low TLR9 expression levels upon diagnosis had significantly shorter breast cancer specific survival, as compared with those patients whose tumors had higher TLR9 protein expression levels.[15] This finding has since been independently verified by a group of French scientists.[32] Furthermore, these findings are not limited to TNBC as low TLR9 expression predicts poor survival also in renal cell carcinoma, pancreatic cancer and possibly also in mucoepidermoid salivary gland carcinoma.[13, 31, 33] Whether low tumor TLR9 expression is only a biomarker for poor survival, or whether it actually contributes to cancer pathophysiology in these tumors, is not currently known. Our studies in pre-clinical cancer models however demonstrated that despite slower *in vitro* growth, TNBC cells with low TLR9 expression formed *in vivo* significantly larger tumors than those with high TLR9 expression.[15] Notably, all our previous (Caucasian) cohorts consisted of patients of European ethnicity, from Northern and Eastern Finland.[15, 33] Ethnicity of the patients with results similar to ours was not disclosed in the French cohort.[32] Despite these promising results, tumor TLR9 expression is not currently used as a prognostic clinical tool. To be used as such will require further clinical studies, and preferably a demonstration that use of the TLR9 biomarker improves patient outcomes.

AA women with TNBC have a worse outcome than Caucasian women. The reason for this health discrepancy has remained unclear and controversial as both socioeconomic factors and tumor biology have been suggested as etiologic factors.[39, 49, 50] To investigate the possible prognostic significance of tumor TLR9 in AA TNBC, we compared tumor TLR9 staining intensity upon diagnosis with disease outcomes in this patient group. Surprisingly, unlike among Caucasian TNBC patient population,[15, 32] high tumor TLR9 protein expression did not protect from relapses among AA TNBC patients. In our previously published EA breast cancer cohort, ~ 90% of TNBC patients with high tumor TLR9 expression survived over 10 years. Of patients in the low TLR9 group, ~ 40% died within the first 5 years.[15] The survival gap between the high and low tumor TLR9 TNBC groups was even greater in the French study.[32] It is currently unclear how high tumor TLR9 expression protects from relapses in various cancers and especially in TNBC. The mechanisms involved could include effects on tumor immunity, tumor invasion, or autophagy. [3, 10, 15, 51] There may however be, SNP-based modifications of the effect of TLR9 on these cellular processes among AA TNBC patients. This could explain the lack of protection that tumor TLR9 provides in other ethnic groups. These issues will, however, require further detailed experimentation at the cellular and molecular level.

In 2003, Lazarus *et al*. reported differences in *TLR9* genetic variation between various ethnic groups.[52] Such differences might result in ethnic-specific TLR9 function or expression patterns that could, ultimately, also contribute towards disease susceptibility, progression, or survival. We therefore carried out a similar but larger effort to understand *TLR9* genetic differences between EAs and AAs. We indeed detected differences. Thirty-two *TLR9* variants had a statistically significant MAF difference between AAs and EAs, most of which have a higher MAF in AAs. Most of the detected differences are predicted to affect CpG-methylation, and thereby, possibly gene expression.[47] Some of the missense variants with statistically significant MAF differences between the ethnicities are predicted to have profound effects on the TLR9 protein and function. Overall, rare missense alleles are more frequently present in AAs compared to EAs; these rare variants could be related to ethnic-specific disease risk. The most notable finding is the MAF distribution of rs352140. This *TLR9* variant has been previously associated with a decreased breast cancer risk.[25] Specifically, the T allele was suggested to be protective of breast cancer in a recent meta-analysis consisting of 12,197 cancer cases and 13,488 controls.[25] We identified a significantly higher T allele frequency in EAs compared to AAs (55.17 vs. 34.52, p = 2.20E-16), suggesting EAs benefit more from the T allele’s protective effects than AAs. This synonymous variant disrupts a CpG site; thus its effect on expression could play a role in disease susceptibility. The mechanism of how the T allele of rs352140 might protect from breast cancer has been investigated, but it is currently debatable.[29, 52–55] In fact, rs352140 has even been associated with an increased risk of certain cancers.[56] Further investigation is required to more clearly understand rs352140 and cancer-specific risk. Noteworthy, through our case/control analysis, we detected even less T alleles in AA breast cancer cases compared to AA controls, which is expected if the allele is protective against breast cancer; however, our results were not statistically significant.

Our efforts to test an association of germline *TLR9* variants with AA breast cancer risk did not reveal any variant class differences between cases and controls; however, three previously unreported *TLR9* variants appears to be slightly associated with late (>45 years) onset breast cancer. These included two missense (p.(D864N) and p.(E865K)) and one synonymous (p.(F873 =)) variants and are predicted to be benign. Furthermore, only the synonymous variant affects a CpG site. Each variant was detected in one breast cancer case in the cohort; thus, these findings need to be replicated. Furthermore, our AA breast cancer cohort did not specifically address TNBC cases, due to limited clinical information available for TCGA data. Therefore, genetic associations specifically with TNBC are warranted. In addition to assessing breast cancer risk, also the contribution of these SNPs to breast cancer specific-survival requires further studies.

It is currently unclear what molecular events drive the expression of the TLR9 variants. Especially the rs352140 variant has been, however, associated with infections such as malaria and meningitis. [53, 57] Specifically, the minor T allele has been associated with increased inflammation and thereby increased symptoms of these infectious diseases, possibly indicating a stronger immune reaction, as also noted in placental inflammation.[29, 58] Whether or not such increased inflammatory response also protects from the development of breast cancer remains to be characterized. Conversely, the weaker immune response, and thereby lesser symptoms may have initially provided an evolutionary survival advantage, possibly explaining the enrichment of such variant in people with African ancestry. Taken together, these hypotheses require further studies.

In conclusion, unlike among Caucasian TNBC patients, high tumor TLR9 protein expression is not associated with improved survival among AA TNBC patients. This may be due to differences in TLR9 function or expression, caused by variants in the *TLR9* gene. Our results show that EAs more frequently carry the T allele of rs352140 when compared to AAs, which has been associated with protection from breast cancer. Thus, this *TLR9* variant may be a previously unknown source of health disparity in breast cancer. Our results need further confirmation, especially in TNBC cohorts. They also call for more in depth studies on the molecular mechanisms of how TLR9 could affect breast cancer development and treatment responses.

## Supporting information

S1 Table Immunohistochemical TLR9 staining scores and the associated clinical data for AA TNBC patients. (XLSX)

S2 Table Clinical and sequencing file information of the 131 AA TCGA breast cancer cases. (DOCX)

S3 Table Descriptive and clinico-pathological parameters of triple-negative breast cancer patients. (DOCX)

S4 Table Genotypes and MAFs of TLR9 germline coding variants detected in AA BC-affected individuals in the TCGA and population controls. (DOCX)
