# Supplementary material for: Lower frequency of TLR9 variant associated with protection from breast cancer among African Americans
Source: PLoS One. 2017 Sep 8;12(9):e0183832. doi: 10.1371/journal.pone.0183832 (PMC5590816; doi:10.1371/journal.pone.0183832)
Supplement: S2 Table — (DOCX) [file pone.0183832.s002.docx]

**S2 Table: Clinical and sequencing file information of the 131 AA TCGA breast cancer cases.**

| Name on VCF file | BAM file name | Case UUID | Patient breast cancer details | | | | | | |
| --- | --- | --- | --- | --- | --- | --- | --- | --- | --- |
|  |  |  | Age of onset | | Classification of tumor | Tumor grade | Tumor stage | Morphology | Family history |
|  |  |  | Years | Days |  |  |  |  |  |
| H_LS-A7-A13D-10A-02D-A272-09 | 04ce584b3adfd3e8e64618b271cb91a8_gdc_realn.bam | [8785012f-f73e-4d68-87cf-1d804af32782](https://gdc-portal.nci.nih.gov/cases/8785012f-f73e-4d68-87cf-1d804af32782) | 46 | 269 | Not reported | Not reported | stage iia | Infiltrating duct carcinoma, NOS | Not reported |
| H_LS-EW-A6SA-10A-01D-A32I-09 | 0743c83fe0068b73fbc5085b760dd9b3_gdc_realn.bam | [aba5f46a-e67a-4cd2-9c52-c0686968ff04](https://gdc-portal.nci.nih.gov/cases/aba5f46a-e67a-4cd2-9c52-c0686968ff04) | 59 | 74 | Not reported | Not reported | stage ii | Infiltrating duct carcinoma, NOS | Not reported |
| H_LS-A2-A259-10A-01D-A16D-09 | 09366ccf12d97c442619020ee8b7c994_gdc_realn.bam | [3fc3755d-a3f8-4e2c-813f-ff124f2a75c1](https://gdc-portal.nci.nih.gov/cases/3fc3755d-a3f8-4e2c-813f-ff124f2a75c1) | 70 | 99 | Not reported | Not reported | stage i | Infiltrating duct carcinoma, NOS | Not reported |
| H_LS-GM-A3XL-10A-01D-A22X-09 | 09d9b143bb0c1e0c3549ac12d2d6c3ad_gdc_realn.bam | [65cac997-4d39-4501-85ec-4fcb328a8eb5](https://gdc-portal.nci.nih.gov/cases/65cac997-4d39-4501-85ec-4fcb328a8eb5) | 49 | 274 | Not reported | Not reported | stage iia | Infiltrating duct carcinoma, NOS | Not reported |
| H_LS-S3-AA10-10A-01D-A41F-09 | 12098276d8dd459ba0838426aadcfc8e_gdc_realn.bam | [23c31c2e-336c-4878-a476-cf8d811b4875](https://gdc-portal.nci.nih.gov/cases/23c31c2e-336c-4878-a476-cf8d811b4875) | 65 | 334 | Not reported | Not reported | stage iia | Infiltrating duct carcinoma, NOS | Not reported |
| H_LS-OL-A5RZ-10A-01D-A28E-09 | 13cc0f00197dedabbc59a7d1231e7d92_gdc_realn.bam | [7e8713bd-0b17-4f09-9696-bc29707d862b](https://gdc-portal.nci.nih.gov/cases/7e8713bd-0b17-4f09-9696-bc29707d862b) | 57 | 219 | Not reported | Not reported | stage ia | Infiltrating duct carcinoma, NOS | Not reported |
| H_LS-E2-A573-10A-01D-A29N-09 | 13da7faefbb48a7350d102c7d6812156_gdc_realn.bam | [6429c443-8ac3-407f-bb9c-66420b904bbf](https://gdc-portal.nci.nih.gov/cases/6429c443-8ac3-407f-bb9c-66420b904bbf) | 48 | 157 | Not reported | Not reported | stage ia | Infiltrating duct carcinoma, NOS | Not reported |
| H_LS-S3-AA12-10A-01D-A41F-09 | 199ddd1943d83fd07877b9d3341fd2c2_gdc_realn.bam | [2bcacfd1-6a45-4b96-9b80-2ad569b1ab45](https://gdc-portal.nci.nih.gov/cases/2bcacfd1-6a45-4b96-9b80-2ad569b1ab45) | 82 | 151 | Not reported | Not reported | stage iiia | Infiltrating duct carcinoma, NOS | Not reported |
| H_LS-EW-A6SD-10A-01D-A33H-09 | 1a776f0afcb96ff80992f96a4c628952_gdc_realn.bam | [42378dbc-a123-4cf9-8502-165e89fda271](https://gdc-portal.nci.nih.gov/cases/42378dbc-a123-4cf9-8502-165e89fda271) | 32 | 318 | Not reported | Not reported | stage iib | Infiltrating duct carcinoma, NOS | Not reported |
| H_LS-A2-A3XV-10A-01D-A23C-09 | 1bbd9fc5e3a99717c592962dd3746305_gdc_realn.bam | [189e1f27-7738-413a-a4d4-97d41d592a13](https://gdc-portal.nci.nih.gov/cases/189e1f27-7738-413a-a4d4-97d41d592a13) | 46 | 170 | Not reported | Not reported | stage iia | Infiltrating duct carcinoma, NOS | Not reported |
| H_LS-AC-A5EH-10A-01D-A28E-09 | 277a10354a27e6e462c253bd56116372_gdc_realn.bam | [d13fb44b-291b-4ea4-920c-142daa8d1989](https://gdc-portal.nci.nih.gov/cases/d13fb44b-291b-4ea4-920c-142daa8d1989) | 76 | 205 | Not reported | Not reported | stage iib | Infiltrating duct carcinoma, NOS | Not reported |
| H_LS-EW-A1PB-10A-01D-A142-09 | 2c083cc0b2334235701e08d7d8c92a44_gdc_realn.bam | [88db1340-e4bf-451a-87c0-6e9168296f5e](https://gdc-portal.nci.nih.gov/cases/88db1340-e4bf-451a-87c0-6e9168296f5e) | 70 | 203 | Not reported | Not reported | stage iiia | Infiltrating duct carcinoma, NOS | Not reported |
| H_LS-LL-A73Y-10B-01D-A33H-09 | 2cefc395ce7e81ae6a2158db0f12cbcf_gdc_realn.bam | [ea645243-df49-4466-a255-9f3d4321e357](https://gdc-portal.nci.nih.gov/cases/ea645243-df49-4466-a255-9f3d4321e357) | 67 | 176 | Not reported | Not reported | stage ia | Infiltrating duct carcinoma, NOS | Not reported |
| H_LS-S3-AA14-10A-01D-A41F-09 | 2f0bfab542e30f66c5babdfcaca6bfa0_gdc_realn.bam | [929acc67-b8bf-4f65-9110-380e44ab66b7](https://gdc-portal.nci.nih.gov/cases/929acc67-b8bf-4f65-9110-380e44ab66b7) | 47 | 186 | Not reported | Not reported | stage i | Infiltrating duct carcinoma, NOS | Not reported |
| H_LS-GM-A2DF-10C-01D-A17W-09 | 36329e007558820988c50246b14eb40c_gdc_realn.bam | [d6f7afc0-1558-43ad-acb1-2b5311ed2264](https://gdc-portal.nci.nih.gov/cases/d6f7afc0-1558-43ad-acb1-2b5311ed2264) | 53 | 256 | Not reported | Not reported | stage iia | Infiltrating duct carcinoma, NOS | Not reported |
| H_LS-AQ-A54O-10A-01D-A25Q-09 | 36e935f7179fc4990ad3d5715e8baac6_gdc_realn.bam | [35ca5e2a-861a-4dd2-a4ce-d294cf080da3](https://gdc-portal.nci.nih.gov/cases/35ca5e2a-861a-4dd2-a4ce-d294cf080da3) | 51 | 67 | Not reported | Not reported | stage iia | Infiltrating duct carcinoma, NOS | Not reported |
| H_LS-UL-AAZ6-10A-01D-A41F-09 | 3a12faf85630d55a774d8b1a60e364dc_gdc_realn.bam | [4b54e06e-a280-4981-a4e1-9aea154341b4](https://gdc-portal.nci.nih.gov/cases/4b54e06e-a280-4981-a4e1-9aea154341b4) | 73 | 336 | Not reported | Not reported | stage iia | Infiltrating duct carcinoma, NOS | Not reported |
| H_LS-A2-A25E-10A-01D-A167-09 | 3cece334b34c6b8641997ff1973dc31c_gdc_realn.bam | [ba89cb4f-cd63-47b1-9550-76fdaff89f2e](https://gdc-portal.nci.nih.gov/cases/ba89cb4f-cd63-47b1-9550-76fdaff89f2e) | 34 | 183 | Not reported | Not reported | stage iiia | Infiltrating duct carcinoma, NOS | Not reported |
| H_LS-A7-A4SD-10A-01D-A25Q-09 | 3e46b6e81ed27fd7dcd8ab6efcb2e524_gdc_realn.bam | [8c09f413-e938-4f2e-a414-84f0e7fcfe41](https://gdc-portal.nci.nih.gov/cases/8c09f413-e938-4f2e-a414-84f0e7fcfe41) | 52 | 138 | Not reported | Not reported | stage iia | Infiltrating duct carcinoma, NOS | Not reported |
| H_LS-OL-A5D7-10A-01D-A27P-09 | 3e72180bf3cddc590fd84eb3da160bf6_gdc_realn.bam | [c8f39325-5382-447c-9291-a6915fc978b8](https://gdc-portal.nci.nih.gov/cases/c8f39325-5382-447c-9291-a6915fc978b8) | 70 | 76 | Not reported | Not reported | stage iia | Infiltrating duct carcinoma, NOS | Not reported |
| H_LS-BH-A5IZ-10A-01D-A27P-09 | 3f2ed206f9d5f4533ec238dfa85594ac_gdc_realn.bam | [a8a33ba9-0976-4e19-b0e9-fa5ba4d4eaa8](https://gdc-portal.nci.nih.gov/cases/a8a33ba9-0976-4e19-b0e9-fa5ba4d4eaa8) | 51 | 300 | Not reported | Not reported | stage iib | Infiltrating duct carcinoma, NOS | Not reported |
| H_LS-AC-A7VB-10A-01D-A351-09 | 3fd11399c67c9e2dd53f29cb51c9e943_gdc_realn.bam | [56230e4a-29ca-4f37-a0f9-730dbdfb6fa2](https://gdc-portal.nci.nih.gov/cases/56230e4a-29ca-4f37-a0f9-730dbdfb6fa2) | 51 | 179 | Not reported | Not reported | stage iia | Infiltrating duct carcinoma, NOS | Not reported |
| H_LS-AC-A3BB-10A-01D-A19Y-09 | 4471d33e50ec78104c1b98a5e3566f7a_gdc_realn.bam | [048549cf-d0a5-4743-a0a1-2004f7cc2b08](https://gdc-portal.nci.nih.gov/cases/048549cf-d0a5-4743-a0a1-2004f7cc2b08) | 46 | 182 | Not reported | Not reported | stage iiia | Lobular carcinoma, NOS | Not reported |
| H_LS-AC-A62X-10A-01D-A29N-09 | 487c40197bc0af2956d0164f31ca3940_gdc_realn.bam | [5b2a4f11-ca46-4974-9420-59b4820920bf](https://gdc-portal.nci.nih.gov/cases/5b2a4f11-ca46-4974-9420-59b4820920bf) | 72 | 329 | Not reported | Not reported | stage iia | Infiltrating duct carcinoma, NOS | Not reported |
| H_LS-A2-A3XY-10A-01D-A23C-09 | 498a64c0e5e32bebe7715bbdc4151983_gdc_realn.bam | [deba32e4-0e68-4711-941b-3b63bd965afb](https://gdc-portal.nci.nih.gov/cases/deba32e4-0e68-4711-941b-3b63bd965afb) | 49 | 162 | Not reported | Not reported | stage iib | Infiltrating duct carcinoma, NOS | Not reported |
| H_LS-S3-AA11-10B-01D-A41F-09 | 4c54d8b7fc3e75ce2aede0256c7783e1_gdc_realn.bam | [0dd8dbc1-c48b-4e7c-b401-57101f724967](https://gdc-portal.nci.nih.gov/cases/0dd8dbc1-c48b-4e7c-b401-57101f724967) | 67 | 342 | Not reported | Not reported | stage iia | Infiltrating duct carcinoma, NOS | Not reported |
| H_LS-BH-A5J0-10A-01D-A27P-09 | 4ca867228ad679e8545a1005d7406dc4_gdc_realn.bam | [db55d9d9-dd56-4ca9-b1f1-0d929697b280](https://gdc-portal.nci.nih.gov/cases/db55d9d9-dd56-4ca9-b1f1-0d929697b280) | 63 | 62 | Not reported | Not reported | stage ia | Infiltrating duct carcinoma, NOS | Not reported |
| H_LS-OL-A5RV-10A-01D-A28E-09 | 4cbc588badaa0793f3c6a0274e83d745_gdc_realn.bam | [1d38d356-d126-4476-94d0-26616b9375b1](https://gdc-portal.nci.nih.gov/cases/1d38d356-d126-4476-94d0-26616b9375b1) | 43 | 115 | Not reported | Not reported | stage iiic | Infiltrating duct carcinoma, NOS | Not reported |
| H_LS-LL-A5YN-10A-01D-A28E-09 | 4cfe9834590e107e24ba3e40a762b767_gdc_realn.bam | [ef1b3332-cd7f-41bb-a2d3-2538e7becc5c](https://gdc-portal.nci.nih.gov/cases/ef1b3332-cd7f-41bb-a2d3-2538e7becc5c) | 46 | 83 | Not reported | Not reported | stage iia | Infiltrating duct carcinoma, NOS | Not reported |
| H_LS-A7-A6VW-10A-01D-A33H-09 | 4d3da6ab58d89b00355fbc71ad65d0cf_gdc_realn.bam | [98e709e7-e195-4b37-9537-f6081affb609](https://gdc-portal.nci.nih.gov/cases/98e709e7-e195-4b37-9537-f6081affb609) | 48 | 7 | Not reported | Not reported | stage iia | Infiltrating duct carcinoma, NOS | Not reported |
| H_LS-A2-A3XW-10A-01D-A23C-09 | 4e63f878852857fd9bd8e89397b5c266_gdc_realn.bam | [c02699b5-580a-4d55-971e-f4692b2cce57](https://gdc-portal.nci.nih.gov/cases/c02699b5-580a-4d55-971e-f4692b2cce57) | 42 | 330 | Not reported | Not reported | stage iib | Infiltrating duct carcinoma, NOS | Not reported |
| H_LS-AC-A3W5-10A-01D-A22A-09 | 4eeeead7425e0ee460896bfba8103565_gdc_realn.bam | [4c313e5b-4dc3-408e-8e92-22427f74743e](https://gdc-portal.nci.nih.gov/cases/4c313e5b-4dc3-408e-8e92-22427f74743e) | 65 | 288 | Not reported | Not reported | stage iia | Lobular carcinoma, NOS | Not reported |
| H_LS-A7-A6VV-10A-01D-A33H-09 | 52b05f72235f8ae9467f618bd29a6d99_gdc_realn.bam | [57af5c72-0d60-4a6b-b1b4-ec6dab90f80f](https://gdc-portal.nci.nih.gov/cases/57af5c72-0d60-4a6b-b1b4-ec6dab90f80f) | 51 | 68 | Not reported | Not reported | stage iia | Infiltrating duct carcinoma, NOS | Not reported |
| H_LS-OL-A6VO-10A-01D-A33H-09 | 594eeade168e5d64dc6127ddef26823b_gdc_realn.bam | [398fb71b-ca83-44e7-bf0d-b1ca464b0283](https://gdc-portal.nci.nih.gov/cases/398fb71b-ca83-44e7-bf0d-b1ca464b0283) | 43 | 75 | Not reported | Not reported | stage ia | Infiltrating duct carcinoma, NOS | Not reported |
| H_LS-BH-A6R8-10A-01D-A33H-09 | 59723bcdd11540ba96a5fe9b12dbddd2_gdc_realn.bam | [91b09291-5944-4691-a0d8-7d1ccc649b9b](https://gdc-portal.nci.nih.gov/cases/91b09291-5944-4691-a0d8-7d1ccc649b9b) | 46 | 28 | Not reported | Not reported | stage iia | Infiltrating duct carcinoma, NOS | Not reported |
| H_LS-A2-A3XT-10A-01D-A22X-09 | 5c47c4dd7c888539466b9f6dc0a6427f_gdc_realn.bam | [c72cb184-462d-4009-9cdb-848782ff8a76](https://gdc-portal.nci.nih.gov/cases/c72cb184-462d-4009-9cdb-848782ff8a76) | 45 | 213 | Not reported | Not reported | stage iib | Infiltrating duct carcinoma, NOS | Not reported |
| H_LS-A2-A4RX-10A-01D-A25Q-09 | 5c852858bd1c6f373e99673fd0a88caa_gdc_realn.bam | [d111c4ab-b39a-4a7c-8d8e-cb342bc10627](https://gdc-portal.nci.nih.gov/cases/d111c4ab-b39a-4a7c-8d8e-cb342bc10627) | 67 | 308 | Not reported | Not reported | stage iia | Metaplastic carcinoma, NOS | Not reported |
| H_LS-OL-A5RX-10A-01D-A28E-09 | 5c9ed4cc1862cfb444f5150979c8f22d_gdc_realn.bam | [43c0bec6-6fac-47b1-83cb-200a15a932be](https://gdc-portal.nci.nih.gov/cases/43c0bec6-6fac-47b1-83cb-200a15a932be) | 51 | 315 | Not reported | Not reported | stage ia | Infiltrating duct carcinoma, NOS | Not reported |
| H_LS-B6-A0I1-10A-01D-A21Q-09 | 5e961411944f0b5763f2c3a1c522157a_gdc_realn.bam | [5dd423e8-feaa-4568-a750-500948c41d6c](https://gdc-portal.nci.nih.gov/cases/5dd423e8-feaa-4568-a750-500948c41d6c) | 73 | 239 | Not reported | Not reported | stage iia | Infiltrating duct carcinoma, NOS | Not reported |
| H_LS-3C-AALJ-10A-01D-A41F-09 | 5fef2b528ec399d5481bfd713ae3940e_gdc_realn.bam | [427d0648-3f77-4ffc-b52c-89855426d647](https://gdc-portal.nci.nih.gov/cases/427d0648-3f77-4ffc-b52c-89855426d647) | 62 | 203 | Not reported | Not reported | stage iib | Infiltrating duct carcinoma, NOS | Not reported |
| H_LS-3C-AALI-10A-01D-A41F-09 | 60a387f0b4ccd48b61eaac978f167e96_gdc_realn.bam | [55262fcb-1b01-4480-b322-36570430c917](https://gdc-portal.nci.nih.gov/cases/55262fcb-1b01-4480-b322-36570430c917) | 50 | 276 | Not reported | Not reported | stage iib | Infiltrating duct carcinoma, NOS | Not reported |
| H_LS-AQ-A54N-10A-01D-A25Q-09 | 6555a1034e73761029d3ffc587d0c57e_gdc_realn.bam | [58090433-d8d1-4499-a235-810c3e9d5b74](https://gdc-portal.nci.nih.gov/cases/58090433-d8d1-4499-a235-810c3e9d5b74) | 51 | 114 | Not reported | Not reported | stage iia | Infiltrating duct carcinoma, NOS | Not reported |
| H_LS-EW-A1J2-10A-01W-A14R-09 | 6cd19796a9cf1724a42c6171bcfbd1e8_gdc_realn.bam | [a381275f-8858-4793-a8a2-0ca9db7274c8](https://gdc-portal.nci.nih.gov/cases/a381275f-8858-4793-a8a2-0ca9db7274c8) | 50 | 296 | Not reported | Not reported | Not reported | Lobular carcinoma, NOS | Not reported |
| H_LS-AC-A8OQ-10A-01D-A41F-09 | 6d7e469475fa2df40cb674edd142927b_gdc_realn.bam | [dfaabd03-2d40-4422-b210-caf112ff4229](https://gdc-portal.nci.nih.gov/cases/dfaabd03-2d40-4422-b210-caf112ff4229) | 72 | 237 | Not reported | Not reported | stage iib | Infiltrating duct carcinoma, NOS | Not reported |
| H_LS-A2-A3XU-10A-01D-A22X-09 | 712f130e6451b07d4ec69abba380c7c8_gdc_realn.bam | [a8e7d8a4-bbf0-496b-b387-8e014cfdcea6](https://gdc-portal.nci.nih.gov/cases/a8e7d8a4-bbf0-496b-b387-8e014cfdcea6) | 35 | 263 | Not reported | Not reported | stage iib | Infiltrating duct carcinoma, NOS | Not reported |
| H_LS-A2-A3XS-10A-01D-A22X-09 | 73b12f8aa5793e982b1727fafc552c09_gdc_realn.bam | [a78b04b4-2380-4803-963e-e4e633cd69ab](https://gdc-portal.nci.nih.gov/cases/a78b04b4-2380-4803-963e-e4e633cd69ab) | 62 | 355 | Not reported | Not reported | stage iiia | Infiltrating duct carcinoma, NOS | Not reported |
| H_LS-AC-A6IW-10A-01D-A33H-09 | 73db8f1758ffbe156b65a97bdb4313e2_gdc_realn.bam | [8183f0fb-2303-4d7b-bccd-55e5031fc7df](https://gdc-portal.nci.nih.gov/cases/8183f0fb-2303-4d7b-bccd-55e5031fc7df) | 73 | 233 | Not reported | Not reported | stage iia | Infiltrating duct carcinoma, NOS | Not reported |
| H_LS-S3-AA17-10A-01D-A41F-09 | 74787a79b7009e5857e2b712c9b5d12f_gdc_realn.bam | [49a2fd48-744d-4d88-b9b4-8c778d4f48fd](https://gdc-portal.nci.nih.gov/cases/49a2fd48-744d-4d88-b9b4-8c778d4f48fd) | 64 | 266 | Not reported | Not reported | stage iib | Infiltrating duct carcinoma, NOS | Not reported |
| H_LS-LL-A740-10B-01D-A32I-09 | 74b87c9985655d9f39e5e5db7b148981_gdc_realn.bam | [aa60fa8d-c374-40e0-af2d-b007701e67e3](https://gdc-portal.nci.nih.gov/cases/aa60fa8d-c374-40e0-af2d-b007701e67e3) | 61 | 136 | Not reported | Not reported | stage ia | Infiltrating duct carcinoma, NOS | Not reported |
| H_LS-A2-A25B-10A-01D-A167-09 | 75458ecd4e627e30a66539dea1a530a9_gdc_realn.bam | [eadf8482-e60e-4307-adb7-d5c3b9fa6cae](https://gdc-portal.nci.nih.gov/cases/eadf8482-e60e-4307-adb7-d5c3b9fa6cae) | 39 | 210 | Not reported | Not reported | stage iib | Infiltrating duct carcinoma, NOS | Not reported |
| H_LS-E2-A56Z-10A-01D-A29N-09 | 79db26720910a2aafe78230f6ae712eb_gdc_realn.bam | [45c3975c-d333-4237-93f7-e12dc2451c38](https://gdc-portal.nci.nih.gov/cases/45c3975c-d333-4237-93f7-e12dc2451c38) | 69 | 80 | Not reported | Not reported | stage iib | Infiltrating duct carcinoma, NOS | Not reported |
| H_LS-LL-A5YM-10A-01D-A28E-09 | 7c67eaf77a0005515b964765107fb4ca_gdc_realn.bam | [e9a12df9-024e-4cbc-b6bf-e4e87485fa90](https://gdc-portal.nci.nih.gov/cases/e9a12df9-024e-4cbc-b6bf-e4e87485fa90) | 88 | 336 | Not reported | Not reported | stage iiib | Paget disease and infiltrating duct carcinoma of breast | Not reported |
| H_LS-A2-A3KC-10A-01D-A20S-09 | 821b02260924a65a53aa4d0a5178699c_gdc_realn.bam | [2d29a4ac-98e7-4663-9dd6-5681bc32ac2e](https://gdc-portal.nci.nih.gov/cases/2d29a4ac-98e7-4663-9dd6-5681bc32ac2e) | 55 | 313 | Not reported | Not reported | stage iib | Lobular carcinoma, NOS | Not reported |
| H_LS-OL-A66H-10A-01D-A29N-09 | 8513fb8a197e21097c1ddf2d2161aca7_gdc_realn.bam | [76bd9bb9-9a8b-4a90-bb7c-eafe76472ee4](https://gdc-portal.nci.nih.gov/cases/76bd9bb9-9a8b-4a90-bb7c-eafe76472ee4) | Not reported |  | Not reported | Not reported | stage ib | Mucinous adenocarcinoma | Not reported |
| H_LS-LL-A441-10A-01D-A243-09 | 8772238ae112a0d246d27be8e2152931_gdc_realn.bam | [69fc24fc-cfe2-487a-935f-1a954b30b709](https://gdc-portal.nci.nih.gov/cases/69fc24fc-cfe2-487a-935f-1a954b30b709) | 62 | 127 | Not reported | Not reported | stage ia | Infiltrating duct carcinoma, NOS | Not reported |
| H_LS-EW-A6S9-10A-01D-A33H-09 | 8d0e9a9a2971e4c11020c55e8037cc89_gdc_realn.bam | [9676e46b-8f29-43cd-ab65-eb6a535cced4](https://gdc-portal.nci.nih.gov/cases/9676e46b-8f29-43cd-ab65-eb6a535cced4) | 34 | 337 | Not reported | Not reported | stage iia | Infiltrating duct carcinoma, NOS | Not reported |
| H_LS-B6-A3ZX-10A-01D-A23C-09 | 8fef34dbc45f141e733cc36b3b448c82_gdc_realn.bam | [9434687a-197c-4959-b6b8-9c05f1dd7f53](https://gdc-portal.nci.nih.gov/cases/9434687a-197c-4959-b6b8-9c05f1dd7f53) | 50 | 172 | Not reported | Not reported | stage iv | Infiltrating duct and lobular carcinoma | Not reported |
| H_LS-5T-A9QA-10A-01D-A41F-09 | 90d0ce7ef03b156c919129c4fa8e0280_gdc_realn.bam | [2fd36838-5a83-433e-ac80-b1f77448e5aa](https://gdc-portal.nci.nih.gov/cases/2fd36838-5a83-433e-ac80-b1f77448e5aa) | 52 | 38 | Not reported | Not reported | stage iia | Infiltrating duct mixed with other types of carcinoma | Not reported |
| H_LS-A7-A5ZW-10A-01D-A29N-09 | 98ac9b132abcc66f2d071f4c289f9f3c_gdc_realn.bam | [523e24a2-51b9-4658-be2f-42e5fccebb17](https://gdc-portal.nci.nih.gov/cases/523e24a2-51b9-4658-be2f-42e5fccebb17) | 47 | 363 | Not reported | Not reported | stage iia | Infiltrating duct carcinoma, NOS | Not reported |
| H_LS-OL-A5D6-10A-01D-A27P-09 | 9a6354376b31ccd5e1e0986128ae898c_gdc_realn.bam | [c3a981c7-f148-4252-bd50-af8a49ec0df8](https://gdc-portal.nci.nih.gov/cases/c3a981c7-f148-4252-bd50-af8a49ec0df8) | 71 | 120 | Not reported | Not reported | stage iia | Infiltrating duct carcinoma, NOS | Not reported |
| H_LS-AC-A62V-10A-01D-A31U-09 | 9b58ef5a029ddf664eb8b4270287fe58_gdc_realn.bam | [d5f2b85a-94a9-4168-bbe8-149ec71342b0](https://gdc-portal.nci.nih.gov/cases/d5f2b85a-94a9-4168-bbe8-149ec71342b0) | 58 | 208 | Not reported | Not reported | stage iv | Infiltrating duct carcinoma, NOS | Not reported |
| H_LS-OL-A5S0-10A-01D-A28E-09 | a2fc99016090c8391527ac5a8f668b95_gdc_realn.bam | [43b3bf47-7313-4dfb-952a-ec0b9d48e16c](https://gdc-portal.nci.nih.gov/cases/43b3bf47-7313-4dfb-952a-ec0b9d48e16c) | 66 | 33 | Not reported | Not reported | stage iib | Infiltrating duct carcinoma, NOS | Not reported |
| H_LS-BH-A42T-10A-01D-A243-09 | a3749b41c77fa57415fa83feec342dba_gdc_realn.bam | [10c829ec-fd66-49c4-8afe-ad3ae567372c](https://gdc-portal.nci.nih.gov/cases/10c829ec-fd66-49c4-8afe-ad3ae567372c) | 75 | 338 | Not reported | Not reported | stage iia | Lobular carcinoma, NOS | Not reported |
| H_LS-LD-A9QF-10A-01D-A41F-09 | aa1851ffb97eca1a3963d2dd5a31eab5_gdc_realn.bam | [95c53ecf-d8f1-4bcb-9b1a-c9a0542939f0](https://gdc-portal.nci.nih.gov/cases/95c53ecf-d8f1-4bcb-9b1a-c9a0542939f0) | 73 | 206 | Not reported | Not reported | stage ia | Infiltrating duct carcinoma, NOS | Not reported |
| H_LS-A7-A6VX-10A-01D-A33H-09 | b079b45a43d7f7499fdb4f6dac9010d3_gdc_realn.bam | [7eea2b6e-771f-44c0-9350-38f45c8dbe87](https://gdc-portal.nci.nih.gov/cases/7eea2b6e-771f-44c0-9350-38f45c8dbe87) | 68 | 229 | Not reported | Not reported | stage iia | Infiltrating duct carcinoma, NOS | Not reported |
| H_LS-A2-A3XX-10A-01D-A23C-09 | b40998d4778f18ed80d6dd8bff0eb761_gdc_realn.bam | [53886143-c1c6-40e9-88e6-e4e5e0271fc8](https://gdc-portal.nci.nih.gov/cases/53886143-c1c6-40e9-88e6-e4e5e0271fc8) | 49 | 84 | Not reported | Not reported | stage iia | Infiltrating duct carcinoma, NOS | Not reported |
| H_LS-GM-A2DO-10D-01D-A18P-09 | ba92beecb9cabcd492f3fcc851aa48c6_gdc_realn.bam | [5fd37868-4762-4109-9dcf-6fdbab5b645d](https://gdc-portal.nci.nih.gov/cases/5fd37868-4762-4109-9dcf-6fdbab5b645d) | 54 | 119 | Not reported | Not reported | stage i | Lobular carcinoma, NOS | Not reported |
| H_LS-LL-A5YO-10A-01D-A28E-09 | bc932fe2bbdee588173d3493f9341201_gdc_realn.bam | [b8aefc48-4a6e-4254-a57f-5f688399b582](https://gdc-portal.nci.nih.gov/cases/b8aefc48-4a6e-4254-a57f-5f688399b582) | 50 | 92 | Not reported | Not reported | stage ia | Infiltrating duct carcinoma, NOS | Not reported |
| H_LS-B6-A409-10A-01D-A243-09 | bcc34fb23f9da70d2d91e2c15c0f76f3_gdc_realn.bam | [5362cbc4-cd28-4afd-a8ac-1a4c2c33cad2](https://gdc-portal.nci.nih.gov/cases/5362cbc4-cd28-4afd-a8ac-1a4c2c33cad2) | 44 | 107 | Not reported | Not reported | stage iiia | Infiltrating duct carcinoma, NOS | Not reported |
| H_LS-OL-A5RY-10A-01D-A28E-09 | bd202014e6ff02cb8ba96e45d81e568b_gdc_realn.bam | [8360cec6-daf6-41c9-9a4f-7fc03c958dcc](https://gdc-portal.nci.nih.gov/cases/8360cec6-daf6-41c9-9a4f-7fc03c958dcc) | 52 | 223 | Not reported | Not reported | stage iia | Infiltrating duct carcinoma, NOS | Not reported |
| H_LS-OL-A6VR-10A-01D-A33H-09 | c05ee0c7d0b22f7f13708a698a89d231_gdc_realn.bam | [db3e1ce8-4a05-4bfc-b5ae-13cf789369e1](https://gdc-portal.nci.nih.gov/cases/db3e1ce8-4a05-4bfc-b5ae-13cf789369e1) | 48 | 170 | Not reported | Not reported | stage ia | Infiltrating duct carcinoma, NOS | Not reported |
| H_LS-LL-A5YP-10A-01D-A28E-09 | c42a5200e73f219a43e4c997f9afe7fb_gdc_realn.bam | [6d24e675-2fa4-4102-861f-7516650b3049](https://gdc-portal.nci.nih.gov/cases/6d24e675-2fa4-4102-861f-7516650b3049) | 49 | 144 | Not reported | Not reported | stage iib | Infiltrating duct carcinoma, NOS | Not reported |
| H_LS-B6-A402-10A-01D-A23C-09 | c57c0d36cd0ea2c1be30fdcf1b50bb58_gdc_realn.bam | [75b3fe55-1a63-426e-867e-2ef52f54778d](https://gdc-portal.nci.nih.gov/cases/75b3fe55-1a63-426e-867e-2ef52f54778d) | 47 | 148 | Not reported | Not reported | stage i | Infiltrating duct carcinoma, NOS | Not reported |
| H_LS-S3-A6ZG-10B-01D-A32I-09 | c5c5fb73a30c399673112547d2dc1cdc_gdc_realn.bam | [1a679332-30a3-4495-a2e5-39d299e14333](https://gdc-portal.nci.nih.gov/cases/1a679332-30a3-4495-a2e5-39d299e14333) | 71 | 209 | Not reported | Not reported | stage iib | Lobular carcinoma, NOS | Not reported |
| H_LS-OL-A66O-10A-01D-A31U-09 | c61047b5e4ae38963735fc0a913eff1e_gdc_realn.bam | [a45833e1-7cc9-4273-b408-620f74679a12](https://gdc-portal.nci.nih.gov/cases/a45833e1-7cc9-4273-b408-620f74679a12) | 39 | 28 | Not reported | Not reported | stage iib | Infiltrating duct carcinoma, NOS | Not reported |
| H_LS-LD-A7W5-10A-01D-A351-09 | c66519996119d299ab1953c2dc593b98_gdc_realn.bam | [a06ff6ae-b0dd-4516-a75c-0db71694b46b](https://gdc-portal.nci.nih.gov/cases/a06ff6ae-b0dd-4516-a75c-0db71694b46b) | 52 | 176 | Not reported | Not reported | stage iiic | Infiltrating duct carcinoma, NOS | Not reported |
| H_LS-WT-AB41-10A-01D-A41F-09 | cb28998343a7db13af7e8db82f8a8ff0_gdc_realn.bam | [e7db08a7-b439-4230-8dc4-1b54af4736c4](https://gdc-portal.nci.nih.gov/cases/e7db08a7-b439-4230-8dc4-1b54af4736c4) | Not reported |  | Not reported | Not reported | stage iib | Infiltrating duct carcinoma, NOS | Not reported |
| H_LS-LL-A7SZ-10A-01D-A351-09 | cc8312c7c2c21acd687dacb1fc0e0738_gdc_realn.bam | [09a5e9fd-d816-4f8c-baa9-0e40ba607b16](https://gdc-portal.nci.nih.gov/cases/09a5e9fd-d816-4f8c-baa9-0e40ba607b16) | 49 | 264 | Not reported | Not reported | stage iib | Infiltrating duct carcinoma, NOS | Not reported |
| H_LS-E2-A2P5-10B-01D-A19Y-09 | ce0bdf722b0d91a4bf1d0d3375d2f065_gdc_realn.bam | [f0de67ff-8967-4fce-9c12-0e07193285cd](https://gdc-portal.nci.nih.gov/cases/f0de67ff-8967-4fce-9c12-0e07193285cd) | 78 | 132 | Not reported | Not reported | stage iiic | Lobular carcinoma, NOS | Not reported |
| H_LS-A2-A4S3-10A-01D-A25Q-09 | d1d4fd4fff38b37c884b015b94b40e2e_gdc_realn.bam | [418c11e8-2670-48d5-bbf5-95b46bff1201](https://gdc-portal.nci.nih.gov/cases/418c11e8-2670-48d5-bbf5-95b46bff1201) | 59 | 213 | Not reported | Not reported | stage iib | Infiltrating duct carcinoma, NOS | Not reported |
| H_LS-EW-A3U0-10A-01D-A22A-09 | d93a87cc9d2a18786a7d2acd9067204c_gdc_realn.bam | [d9dc3b59-613d-469e-8b4f-6c5a557eb26a](https://gdc-portal.nci.nih.gov/cases/d9dc3b59-613d-469e-8b4f-6c5a557eb26a) | 61 | 240 | Not reported | Not reported | stage iiia | Infiltrating duct carcinoma, NOS | Not reported |
| H_LS-LL-A73Z-10B-01D-A32I-09 | dc3bdc6ecada4ce3a58d90a5a00d9adf_gdc_realn.bam | [3e637872-f5e5-49d3-bb0d-9c16b8713382](https://gdc-portal.nci.nih.gov/cases/3e637872-f5e5-49d3-bb0d-9c16b8713382) | 55 | 30 | Not reported | Not reported | stage iv | Infiltrating duct carcinoma, NOS | Not reported |
| H_LS-LL-A8F5-10A-01D-A36M-09 | dde145df12091f6662d66480293f7ddb_gdc_realn.bam | [e1786e78-ac46-46fd-8ce0-8f7456e46082](https://gdc-portal.nci.nih.gov/cases/e1786e78-ac46-46fd-8ce0-8f7456e46082) | 61 | 264 | Not reported | Not reported | stage iia | Infiltrating duct carcinoma, NOS | Not reported |
| H_LS-A7-A26G-10A-01D-A167-09 | e59dcbdcafe075313b13f2c45c1b6df7_gdc_realn.bam | [5ed024e8-d05e-4c65-9441-eda9930ccc82](https://gdc-portal.nci.nih.gov/cases/5ed024e8-d05e-4c65-9441-eda9930ccc82) | 50 | 100 | Not reported | Not reported | stage iia | Metaplastic carcinoma, NOS | Not reported |
| H_LS-EW-A1OZ-10A-01D-A142-09 | e5f5eba079ea330a6d8000bfb44e30ce_gdc_realn.bam | [8f912a40-3251-4e1f-ae65-1f93d71f9881](https://gdc-portal.nci.nih.gov/cases/8f912a40-3251-4e1f-ae65-1f93d71f9881) | 56 | 210 | Not reported | Not reported | stage ia | Infiltrating duct carcinoma, NOS | Not reported |
| H_LS-B6-A401-10A-01D-A23C-09 | e7c4c0f7b5c392112654c0c63a0ac7cc_gdc_realn.bam | [48ff0420-0f19-4e9d-a75d-6c54949a461a](https://gdc-portal.nci.nih.gov/cases/48ff0420-0f19-4e9d-a75d-6c54949a461a) | 47 | 272 | Not reported | Not reported | stage iia | Infiltrating duct carcinoma, NOS | Not reported |
| H_LS-V7-A7HQ-10A-01D-A33H-09 | e8e64a5af850eb22284e4bcb3454ac91_gdc_realn.bam | [1285eb55-415c-494a-aa58-936f0427cdd0](https://gdc-portal.nci.nih.gov/cases/1285eb55-415c-494a-aa58-936f0427cdd0) | 75 | 291 | Not reported | Not reported | stage iiia | Infiltrating duct carcinoma, NOS | Not reported |
| H_LS-AC-A5XU-10A-01D-A28E-09 | ed933a9a7ac8b3208301ffafdf27af07_gdc_realn.bam | [97677fd7-8523-48c5-a8ce-614fd4010551](https://gdc-portal.nci.nih.gov/cases/97677fd7-8523-48c5-a8ce-614fd4010551) | 74 | 163 | Not reported | Not reported | stage iib | Infiltrating duct carcinoma, NOS | Not reported |
| H_LS-BH-A42U-10A-01D-A243-09 | f40a6abcac148967f32ed3b99c014500_gdc_realn.bam | [2dc1cec9-925a-417f-9e21-3c2143e711b4](https://gdc-portal.nci.nih.gov/cases/2dc1cec9-925a-417f-9e21-3c2143e711b4) | 80 | 110 | Not reported | Not reported | stage iia | Lobular carcinoma, NOS | Not reported |
| H_LS-LL-A6FR-10B-01D-A31U-09 | f4875b7cc99323197ac0214bb4f8a59a_gdc_realn.bam | [1347c23d-8d6e-4c15-9461-640249838c96](https://gdc-portal.nci.nih.gov/cases/1347c23d-8d6e-4c15-9461-640249838c96) | 50 | 258 | Not reported | Not reported | stage iia | Infiltrating duct carcinoma, NOS | Not reported |
| H_LS-E2-A572-10B-01D-A31U-09 | f7d37ae4845f6297efaf64fabc213fab_gdc_realn.bam | [06ef6e83-5583-45b9-a0bd-edea68943083](https://gdc-portal.nci.nih.gov/cases/06ef6e83-5583-45b9-a0bd-edea68943083) | 72 | 101 | Not reported | Not reported | stage iiia | Infiltrating duct carcinoma, NOS | Not reported |
| H_LS-OL-A97C-10A-01D-A41F-09 | f824b4908c3aa0b3ec1a120740bfd2e3_gdc_realn.bam | [6ffd1b8c-6f0b-456a-9160-e0c8021e5897](https://gdc-portal.nci.nih.gov/cases/6ffd1b8c-6f0b-456a-9160-e0c8021e5897) | 67 | 264 | Not reported | Not reported | stage iib | Phyllodes tumor, malignant | Not reported |
| H_LS-A7-A2KD-10A-01D-A21Q-09 | f876158f6f7bd2da910b88fd887d753c_gdc_realn.bam | [92654f8d-5137-4ffd-aaa3-5ae38eb88226](https://gdc-portal.nci.nih.gov/cases/92654f8d-5137-4ffd-aaa3-5ae38eb88226) | 53 | 205 | Not reported | Not reported | stage iiia | Infiltrating duct carcinoma, NOS | Not reported |
| H_LS-B6-A400-10A-01D-A23C-09 | fbb2eec91a54bcfed9709c90a392af3d_gdc_realn.bam | [d9a1c06f-7b50-46b0-878e-89e8c31863ae](https://gdc-portal.nci.nih.gov/cases/d9a1c06f-7b50-46b0-878e-89e8c31863ae) | 43 | 317 | Not reported | Not reported | stage iiia | Infiltrating duct carcinoma, NOS | Not reported |
| H_LS-A7-A4SE-10A-01D-A25Q-09 | fbf68f1cad2ea335debf24518e1f9d11_gdc_realn.bam | [dbf6f981-15cc-40ad-91ac-a66360405fbd](https://gdc-portal.nci.nih.gov/cases/dbf6f981-15cc-40ad-91ac-a66360405fbd) | 54 | 357 | Not reported | Not reported | stage iia | Infiltrating duct carcinoma, NOS | Not reported |
| H_LS-EW-A1PC-10A-01D-A21Q-09 | fde9292a36f4eaad364a038049683dae_gdc_realn.bam | [f45210d3-9e66-4f5e-bef1-5ee5547cc893](https://gdc-portal.nci.nih.gov/cases/f45210d3-9e66-4f5e-bef1-5ee5547cc893) | 66 | 328 | Not reported | Not reported | stage iib | Infiltrating duct carcinoma, NOS | Not reported |
| H_LS-OL-A66P-10A-01D-A31U-09 | fe5caeb8815a49752a5cf20c410e2159_gdc_realn.bam | [f81ac8a2-4ce6-439e-b027-1c0bfc88ceaa](https://gdc-portal.nci.nih.gov/cases/f81ac8a2-4ce6-439e-b027-1c0bfc88ceaa) | 75 | 96 | Not reported | Not reported | stage iia | Infiltrating duct carcinoma, NOS | Not reported |
| H_LS-EW-A6SB-10A-01D-A32I-09 | fed68ec520313a3c086bf1ae8f9cad40_gdc_realn.bam | [5c59028f-b8fa-4811-8314-be3eaed5f364](https://gdc-portal.nci.nih.gov/cases/5c59028f-b8fa-4811-8314-be3eaed5f364) | 62 | 199 | Not reported | Not reported | stage ii | Infiltrating duct carcinoma, NOS | Not reported |
| H_LS-A1-A0SJ-10A-02D-A099-09 | TCGA-A1-A0SJ-10A-02D-A099-09_IlluminaGA-DNASeq_exome_gdc_realn.bam | [a2db9dd1-44d5-48b5-817c-a21a85fadb21](https://gdc-portal.nci.nih.gov/cases/a2db9dd1-44d5-48b5-817c-a21a85fadb21) | 39 | 139 | Not reported | Not reported | stage iiia | Infiltrating duct carcinoma, NOS | Not reported |
| H_LS-A2-A04R-10B-01D-A10G-09 | TCGA-A2-A04R-10B-01D-A10G-09_IlluminaGA-DNASeq_exome_HOLD_QC_PENDING_gdc_realn.bam | [f3a058b9-fb7f-4098-8f0e-83c2acfa1ed5](https://gdc-portal.nci.nih.gov/cases/f3a058b9-fb7f-4098-8f0e-83c2acfa1ed5) | 36 | 334 | Not reported | Not reported | stage ia | Infiltrating duct carcinoma, NOS | Not reported |
| H_LS-A2-A0CM-10A-01W-A055-09-1 | TCGA-A2-A0CM-10A-01W-A055-09_IlluminaGA-DNASeq_exome_gdc_realn.bam | [eb2dbb4f-66b6-4525-8323-431970f7a64e](https://gdc-portal.nci.nih.gov/cases/eb2dbb4f-66b6-4525-8323-431970f7a64e) | 40 | 359 | Not reported | Not reported | stage iia | Infiltrating duct carcinoma, NOS | Not reported |
| H_LS-A2-A0CQ-10A-01W-A055-09-1 | TCGA-A2-A0CQ-10A-01W-A055-09_IlluminaGA-DNASeq_exome_gdc_realn.bam | [ab34a9a2-d72d-4106-94fb-118844b1b60b](https://gdc-portal.nci.nih.gov/cases/ab34a9a2-d72d-4106-94fb-118844b1b60b) | 62 | 165 | Not reported | Not reported | stage ia | Infiltrating duct carcinoma, NOS | Not reported |
| H_LS-A2-A0D0-10A-01W-A021-09-1 | TCGA-A2-A0D0-10A-01W-A021-09_IlluminaGA-DNASeq_exome_2_gdc_realn.bam | [324bcba2-f6a4-45a6-807c-215bdffcca21](https://gdc-portal.nci.nih.gov/cases/324bcba2-f6a4-45a6-807c-215bdffcca21) | 60 | 200 | Not reported | Not reported | stage iia | Infiltrating duct carcinoma, NOS | Not reported |
| H_LS-A2-A0D4-10A-01W-A021-09-1 | TCGA-A2-A0D4-10A-01W-A021-09_IlluminaGA-DNASeq_exome_gdc_realn.bam | [75113445-d2d6-44a0-866c-c9175e6d214b](https://gdc-portal.nci.nih.gov/cases/75113445-d2d6-44a0-866c-c9175e6d214b) | 37 | 291 | Not reported | Not reported | stage iib | Infiltrating duct carcinoma, NOS | Not reported |
| H_LS-A2-A0EQ-10A-01W-A055-09-1 | TCGA-A2-A0EQ-10A-01W-A055-09_IlluminaGA-DNASeq_exome_gdc_realn.bam | [2192c1db-4718-4254-ba42-3ae7f30ad5a8](https://gdc-portal.nci.nih.gov/cases/2192c1db-4718-4254-ba42-3ae7f30ad5a8) | 64 | 167 | Not reported | Not reported | stage iia | Infiltrating duct carcinoma, NOS | Not reported |
| H_LS-A2-A0YF-10A-01D-A10G-09 | TCGA-A2-A0YF-10A-01D-A10G-09_IlluminaGA-DNASeq_exome_HOLD_QC_PENDING_gdc_realn.bam | [ae8c77fe-e6c8-44d5-8265-4a38c637bbef](https://gdc-portal.nci.nih.gov/cases/ae8c77fe-e6c8-44d5-8265-4a38c637bbef) | 67 | 189 | Not reported | Not reported | stage i | Infiltrating duct carcinoma, NOS | Not reported |
| H_LS-A7-A13D-10A-02D-A12Q-09 | TCGA-A7-A13D-10A-02D-A12Q-09_IlluminaGA-DNASeq_exome_gdc_realn.bam | [8785012f-f73e-4d68-87cf-1d804af32782](https://gdc-portal.nci.nih.gov/cases/8785012f-f73e-4d68-87cf-1d804af32782) | 46 | 269 | Not reported | Not reported | stage iia | Infiltrating duct carcinoma, NOS | Not reported |
| H_LS-AO-A03O-10A-01W-A021-09-1 | TCGA-AO-A03O-10A-01W-A021-09_IlluminaGA-DNASeq_exome_gdc_realn.bam | [1111fb30-46ce-4722-98da-ec9ba2323d90](https://gdc-portal.nci.nih.gov/cases/1111fb30-46ce-4722-98da-ec9ba2323d90) | 69 | 160 | Not reported | Not reported | stage iia | Infiltrating duct carcinoma, NOS | Not reported |
| H_LS-AO-A03P-10A-01W-A021-09-1 | TCGA-AO-A03P-10A-01W-A021-09_IlluminaGA-DNASeq_exome_gdc_realn.bam | [2d4c778c-7f77-4f0a-8261-2086accf15fd](https://gdc-portal.nci.nih.gov/cases/2d4c778c-7f77-4f0a-8261-2086accf15fd) | 54 | 1 | Not reported | Not reported | stage iib | Infiltrating duct carcinoma, NOS | Not reported |
| H_LS-AO-A0J4-10A-01W-A055-09-1 | TCGA-AO-A0J4-10A-01W-A055-09_IlluminaGA-DNASeq_exome_gdc_realn.bam | [a9b7d7fe-be31-4f71-afee-c1bfdf511888](https://gdc-portal.nci.nih.gov/cases/a9b7d7fe-be31-4f71-afee-c1bfdf511888) | 41 | 77 | Not reported | Not reported | stage ia | Infiltrating duct carcinoma, NOS | Not reported |
| H_LS-AO-A0JA-10A-01W-A071-09-1 | TCGA-AO-A0JA-10A-01W-A071-09_HOLD_QC_PENDING_IlluminaGA-DNASeq_exome_gdc_realn.bam | [86b7b206-8e69-432d-acc9-bccce710955e](https://gdc-portal.nci.nih.gov/cases/86b7b206-8e69-432d-acc9-bccce710955e) | 36 | 158 | Not reported | Not reported | stage iiic | Infiltrating duct carcinoma, NOS | Not reported |
| H_LS-AO-A12H-10A-01D-A110-09 | TCGA-AO-A12H-10A-01D-A110-09_IlluminaGA-DNASeq_exome_1_gdc_realn.bam | [b5b99291-507e-4b68-a039-9a0f571f55df](https://gdc-portal.nci.nih.gov/cases/b5b99291-507e-4b68-a039-9a0f571f55df) | 69 | 313 | Not reported | Not reported | stage iia | Infiltrating duct mixed with other types of carcinoma | Not reported |
| H_LS-AR-A0TT-10A-01D-A099-09 | TCGA-AR-A0TT-10A-01D-A099-09_IlluminaGA-DNASeq_exome_gdc_realn.bam | [13ca97a1-75bb-4a49-b8c3-181e5b2c9e86](https://gdc-portal.nci.nih.gov/cases/13ca97a1-75bb-4a49-b8c3-181e5b2c9e86) | 53 | 321 | Not reported | Not reported | stage iiia | Infiltrating duct carcinoma, NOS | Not reported |
| H_LS-B6-A0I6-10A-01W-A055-09-1 | TCGA-B6-A0I6-10A-01W-A055-09_IlluminaGA-DNASeq_exome_gdc_realn.bam | [045c13ef-3db7-4adf-b0a3-23338f0479f3](https://gdc-portal.nci.nih.gov/cases/045c13ef-3db7-4adf-b0a3-23338f0479f3) | 49 | 117 | Not reported | Not reported | stage iia | Infiltrating duct carcinoma, NOS | Not reported |
| H_LS-B6-A0IE-10A-01W-A055-09-1 | TCGA-B6-A0IE-10A-01W-A055-09_IlluminaGA-DNASeq_exome_gdc_realn.bam | [3f834fa7-6d7b-4b85-98c0-5c55d55b6c95](https://gdc-portal.nci.nih.gov/cases/3f834fa7-6d7b-4b85-98c0-5c55d55b6c95) | 38 | 103 | Not reported | Not reported | stage iiia | Lobular carcinoma, NOS | Not reported |
| H_LS-B6-A0IJ-10A-01W-A055-09-1 | TCGA-B6-A0IJ-10A-01W-A055-09_IlluminaGA-DNASeq_exome_gdc_realn.bam | [2c86c3ea-d926-4d39-a5ae-39ece4774287](https://gdc-portal.nci.nih.gov/cases/2c86c3ea-d926-4d39-a5ae-39ece4774287) | 42 | 285 | Not reported | Not reported | stage iib | Infiltrating duct carcinoma, NOS | Not reported |
| H_LS-B6-A0IM-10A-01W-A055-09-1 | TCGA-B6-A0IM-10A-01W-A055-09_IlluminaGA-DNASeq_exome_gdc_realn.bam | [5a4b7098-1f6a-49e9-b364-29d6d34f5bd5](https://gdc-portal.nci.nih.gov/cases/5a4b7098-1f6a-49e9-b364-29d6d34f5bd5) | 75 | 164 | Not reported | Not reported | stage iib | Infiltrating duct mixed with other types of carcinoma | Not reported |
| H_LS-B6-A0RE-10A-01W-A071-09-1 | TCGA-B6-A0RE-10A-01W-A071-09_HOLD_QC_PENDING_IlluminaGA-DNASeq_exome_gdc_realn.bam | [08da7c4c-3067-4bcf-9d7a-78566df72e69](https://gdc-portal.nci.nih.gov/cases/08da7c4c-3067-4bcf-9d7a-78566df72e69) | 61 | 169 | Not reported | Not reported | stage x | Infiltrating duct carcinoma, NOS | Not reported |
| H_LS-B6-A0RH-10A-01D-A110-09 | TCGA-B6-A0RH-10A-01D-A110-09_IlluminaGA-DNASeq_exome_gdc_realn.bam | [c3148e68-1739-4334-abda-1dcbc2166846](https://gdc-portal.nci.nih.gov/cases/c3148e68-1739-4334-abda-1dcbc2166846) | 51 | 132 | Not reported | Not reported | stage iia | Infiltrating duct carcinoma, NOS | Not reported |
| H_LS-B6-A0RL-10A-01D-A099-09 | TCGA-B6-A0RL-10A-01D-A099-09_IlluminaGA-DNASeq_exome_gdc_realn.bam | [5562455d-bbb8-46eb-a510-e8c6e1e26789](https://gdc-portal.nci.nih.gov/cases/5562455d-bbb8-46eb-a510-e8c6e1e26789) | 30 | 323 | Not reported | Not reported | stage iia | Infiltrating duct carcinoma, NOS | Not reported |
| H_LS-B6-A0RM-10A-01D-A099-09 | TCGA-B6-A0RM-10A-01D-A099-09_IlluminaGA-DNASeq_exome_gdc_realn.bam | [151f3677-6854-4265-9126-d92c646c67ae](https://gdc-portal.nci.nih.gov/cases/151f3677-6854-4265-9126-d92c646c67ae) | 57 | 228 | Not reported | Not reported | stage x | Infiltrating duct carcinoma, NOS | Not reported |
| H_LS-B6-A0WS-10A-01D-A110-09 | TCGA-B6-A0WS-10A-01D-A110-09_IlluminaGA-DNASeq_exome_gdc_realn.bam | [197741de-a098-4f04-bb37-8ea8b3bb39bc](https://gdc-portal.nci.nih.gov/cases/197741de-a098-4f04-bb37-8ea8b3bb39bc) | 58 | 46 | Not reported | Not reported | stage x | Infiltrating duct carcinoma, NOS | Not reported |
| H_LS-B6-A0WZ-10A-01D-A10G-09 | TCGA-B6-A0WZ-10A-01D-A10G-09_IlluminaGA-DNASeq_exome_HOLD_QC_PENDING_gdc_realn.bam | [08740d7f-5a5e-4dfa-bd48-7fbf228a7a28](https://gdc-portal.nci.nih.gov/cases/08740d7f-5a5e-4dfa-bd48-7fbf228a7a28) | 50 | 322 | Not reported | Not reported | stage ii | Infiltrating duct carcinoma, NOS | Not reported |
| H_LS-B6-A0X5-10A-01D-A10G-09 | TCGA-B6-A0X5-10A-01D-A10G-09_IlluminaGA-DNASeq_exome_2_gdc_realn.bam | [4e6edfe6-adcb-4c12-8ff4-38a79f5887e8](https://gdc-portal.nci.nih.gov/cases/4e6edfe6-adcb-4c12-8ff4-38a79f5887e8) | 61 | 100 | Not reported | Not reported | stage iib | Infiltrating duct carcinoma, NOS | Not reported |
| H_LS-BH-A0AV-10A-01D-A110-09 | TCGA-BH-A0AV-10A-01D-A110-09_IlluminaGA-DNASeq_exome_2_gdc_realn.bam | [790ff5db-b7f3-4946-aaed-305f66b1dd6a](https://gdc-portal.nci.nih.gov/cases/790ff5db-b7f3-4946-aaed-305f66b1dd6a) | 52 | 321 | Not reported | Not reported | stage i | Infiltrating duct carcinoma, NOS | Not reported |
| H_LS-BH-A0B0-10A-01D-A110-09 | TCGA-BH-A0B0-10A-01D-A110-09_IlluminaGA-DNASeq_exome_gdc_realn.bam | [66af34ea-f471-4092-aff6-9ac9a5b75b11](https://gdc-portal.nci.nih.gov/cases/66af34ea-f471-4092-aff6-9ac9a5b75b11) | 56 | 322 | Not reported | Not reported | stage i | Infiltrating duct carcinoma, NOS | Not reported |
| H_LS-BH-A0BW-10A-01D-A110-09 | TCGA-BH-A0BW-10A-01D-A110-09_IlluminaGA-DNASeq_exome_gdc_realn.bam | [5d1d00c6-fcae-479e-ae1e-de76efd41d98](https://gdc-portal.nci.nih.gov/cases/5d1d00c6-fcae-479e-ae1e-de76efd41d98) | 71 | 42 | Not reported | Not reported | stage i | Infiltrating duct carcinoma, NOS | Not reported |
| H_LS-BH-A0E2-10A-01W-A071-09-1 | TCGA-BH-A0E2-10A-01W-A071-09_HOLD_QC_PENDING_IlluminaGA-DNASeq_exome_gdc_realn.bam | [8ace3b8c-f23e-48a9-a137-9e046bd9549b](https://gdc-portal.nci.nih.gov/cases/8ace3b8c-f23e-48a9-a137-9e046bd9549b) | 49 | 255 | Not reported | Not reported | stage iiia | Infiltrating duct carcinoma, NOS | Not reported |
| H_LS-E2-A14X-10A-01D-A110-09 | TCGA-E2-A14X-10A-01D-A110-09_IlluminaGA-DNASeq_exome_1_gdc_realn.bam | [60df7543-6da5-4c75-943b-5800c1e08234](https://gdc-portal.nci.nih.gov/cases/60df7543-6da5-4c75-943b-5800c1e08234) | 55 | 17 | Not reported | Not reported | stage iiia | Infiltrating duct carcinoma, NOS | Not reported |
| H_LS-E2-A15L-10A-01D-A12B-09 | TCGA-E2-A15L-10A-01D-A12B-09_IlluminaGA-DNASeq_exome_gdc_realn.bam | [37242f5a-25ae-4b1f-9ce6-09ce1dc92539](https://gdc-portal.nci.nih.gov/cases/37242f5a-25ae-4b1f-9ce6-09ce1dc92539) | 65 | 104 | Not reported | Not reported | stage iia | Lobular carcinoma, NOS | Not reported |
| H_LS-E2-A15O-10A-01D-A110-09 | TCGA-E2-A15O-10A-01D-A110-09_IlluminaGA-DNASeq_exome_gdc_realn.bam | [eafa45a4-2fd1-4fa3-860c-9d52ed382b7d](https://gdc-portal.nci.nih.gov/cases/eafa45a4-2fd1-4fa3-860c-9d52ed382b7d) | 89 | 76 | Not reported | Not reported | stage i | Infiltrating duct carcinoma, NOS | Not reported |
