# Supplementary material for: Lower frequency of TLR9 variant associated with protection from breast cancer among African Americans
Source: PLoS One. 2017 Sep 8;12(9):e0183832. doi: 10.1371/journal.pone.0183832 (PMC5590816; doi:10.1371/journal.pone.0183832)
Supplement: S3 Table — (DOCX) [file pone.0183832.s003.docx]

| **S3 Table.** **Descriptive and clinico-pathological parameters of triple-negative breast cancer patients.** | |
| --- | --- |
| Characteristic | n (%) |
| Pre-treatment clinical T stage  T1a  T1b  T1c  T2  T3  T4a  T4b  T4c  T4d | 0 (0.0)  2 (4.4)  8 (17.4)  21 (45.6)  8 (17.4)  1 (2.2)  3 (6.5)  0 (0.0)  3 (6.5) |
| Pre-treatment clinical N stage  0  1  2 | 30 (66.7)  11 (24.4)  4 (8.9) |
| Pre-treatment group stage  IA  IB  IIA  IIB  IIIA  IIIB  IIIC | 3 (6.7)  6 (13.3)  16 (35.6)  11 (24.4)  3 (6.7)  6 (13.3)  0 (0.0) |
| Histology  IDC (invasive ductal carcinoma)  ILC (invasive lobular carcinoma)  Mixed  Other | 43 (89.6)  1 (2.1)  2 (4.2)  2 (4.1) |
| Grade  2  3 | 3 (6.5)  43 (93.5) |
| Lymph Vascular Space Invasion  Yes  No | 11 (37.9)  18 (62.1) |
| Surgery type  Lumpectomy  MRM (modified radical mastectomy)  TM (total mastectomy)  SSM (skin-sparing mastectomy) | 18 (39.1)  19 (41.3)  8 (17.4)  1 (2.2) |
| Neoadjuvant chemotherapy  Yes  No | 11 (26.2)  31 (73.8) |
| Adjuvant chemotherapy  Yes  No | 32 (74.4)  11 (25.6) |
| Radiation therapy  Yes  No | 31 (66.0)  16 (34.0) |
| Ipsilateral breast tumor recurrence  Yes  No | 11 (25.0)  33 (75.0) |
| Pattern of first recurrence  Local  Regional  Distant  Mixed | 7 (35.0)  1 (5.0)  9 (45.0)  3 (15.0) |
| Regional recurrence  Yes  No | 5 (11.4)  39 (88.6) |
| Combined local and regional recurrence  Yes  No | 10 (25.0)  30 (75.0) |
| Distant metastasis  Yes  No | 15 (33.3)  30 (66.7) |
|  | Median (range) |
| Age at diagnosis (yrs) | 51.0 (21-91) |
| Follow-up time (yrs) | 3.53 (0.01-12.2) |
| TLR9 | 8.5 (0-16) |
