# Supplementary material for: Lower frequency of TLR9 variant associated with protection from breast cancer among African Americans
Source: PLoS One. 2017 Sep 8;12(9):e0183832. doi: 10.1371/journal.pone.0183832 (PMC5590816; doi:10.1371/journal.pone.0183832)
Supplement: S4 Table — (DOCX) [file pone.0183832.s004.docx]

**S4 Table: Genotypes and MAFs of TLR9 germline coding variants detected in AA BC-affected individuals in the TCGA and population controls.**

| GRCh38 Position | rs ID | Alleles | cDNA Change | Protein Change | PolyPhen2 (Class:Score) | CpG site | TCGA BC cohort (n=131) | | | | | | | | TCGA BC cohort - ≤ 45 years at diagnosis (n=21) | | | | | | | | TCGA BC cohort - > 45 years at diagnosis (n=110) | | | | | | | | EVS AA - population controls | | | | | | | |
| --- | --- | --- | --- | --- | --- | --- | --- | --- | --- | --- | --- | --- | --- | --- | --- | --- | --- | --- | --- | --- | --- | --- | --- | --- | --- | --- | --- | --- | --- | --- | --- | --- | --- | --- | --- | --- | --- | --- |
|  |  |  |  |  |  |  | Genotype | | | | Number of alleles | | | MAF | Genotype | | | | Number of alleles | | | MAF | Genotype | | | | Number of alleles | | | MAF | Genotype | | | | Number of alleles | | | MAF |
|  |  |  |  |  |  |  | Het variant | Homo variant | Homo wt | Total | Minor allele | Major allele | Total |  | Het variant | Homo variant | Homo wt | Total | Minor allele | Major allele | Total |  | Het variant | Homo variant | Homo wt | Total | Minor allele | Major allele | Total |  | Het variant | Homo variant | Homo wt | Total | Minor allele | Major allele | Total |  |
| 3:52224303 | rs5743842 | G>A | c.13C>T | p.(R5C) | benign:0.002 | disrupt | 7 | 0 | 124 | 131 | 7 | 255 | 262 | 2.67 | 2 | 0 | 19 | 21 | 2 | 40 | 42 | 4.76 | 5 | 0 | 105 | 110 | 5 | 215 | 220 | 2.27 | 2 | 161 | 2014 | 2177 | 165 | 4189 | 4354 | 3.79 |
| 3:52224246 | rs146965009 | G>A | c.70C>T | p.(L24=) | unknown | no affect | 2 | 0 | 129 | 131 | 2 | 260 | 262 | 0.76 | 0 | 0 | 21 | 21 | 0 | 42 | 42 | 0.00 | 2 | 0 | 108 | 110 | 2 | 218 | 220 | 0.91 | 11 | 0 | 2191 | 2202 | 11 | 4393 | 4404 | 0.25 |
| 3:52223974 | rs56116373 | G>A | c.342C>T | p.(I114=) | unknown | disrupt | 1 | 0 | 130 | 131 | 1 | 261 | 262 | 0.38 | 0 | 0 | 21 | 21 | 0 | 42 | 42 | 0.00 | 1 | 0 | 109 | 110 | 1 | 219 | 220 | 0.45 | 2 | 0 | 2201 | 2203 | 2 | 4404 | 4406 | 0.05 |
| 3:52223875 | rs116310431 | G>T | c.441C>A | p.(S147=) | unknown | no affect | 4 | 0 | 127 | 131 | 4 | 258 | 262 | 1.53 | 2 | 0 | 19 | 21 | 2 | 40 | 42 | 4.76 | 2 | 0 | 108 | 110 | 2 | 218 | 220 | 0.91 | 99 | 2 | 2102 | 2203 | 103 | 4303 | 4406 | 2.34 |
| 3:52223812 | rs72959313 | G>A | c.504C>T | p.(A168=) | unknown | no affect | 2 | 1 | 128 | 131 | 4 | 258 | 262 | 1.53 | 0 | 0 | 21 | 21 | 0 | 42 | 42 | 0.00 | 2 | 1 | 107 | 110 | 4 | 216 | 220 | 1.82 | 57 | 0 | 2146 | 2203 | 57 | 4349 | 4406 | 1.29 |
| 3:52223791 | rs138035523 | G>A | c.525C>T | p.(D175=) | unknown | disrupt | 2 | 0 | 129 | 131 | 2 | 260 | 262 | 0.76 | 0 | 0 | 21 | 21 | 0 | 42 | 42 | 0.00 | 2 | 0 | 108 | 110 | 2 | 218 | 220 | 0.91 | 15 | 0 | 2188 | 2203 | 15 | 4391 | 4406 | 0.34 |
| 3:52223749 | rs143323734 | C>T | c.567G>A | p.(E189=) | unknown | no affect | 1 | 0 | 130 | 131 | 1 | 261 | 262 | 0.38 | 0 | 0 | 21 | 21 | 0 | 42 | 42 | 0.00 | 1 | 0 | 109 | 110 | 1 | 219 | 220 | 0.45 | 2 | 0 | 2201 | 2203 | 2 | 4404 | 4406 | 0.05 |
| 3:52223619 | rs137890561 | C>T | c.697G>A | p.(V233I) | benign:0.011 | disrupt | 1 | 0 | 130 | 131 | 1 | 261 | 262 | 0.38 | 0 | 0 | 21 | 21 | 0 | 42 | 42 | 0.00 | 1 | 0 | 109 | 110 | 1 | 219 | 220 | 0.45 | 2 | 0 | 2201 | 2203 | 2 | 4404 | 4406 | 0.05 |
| 3:52223569 | rs140856643 | G>A | c.747C>T | p.(L249=) | unknown | disrupt | 1 | 0 | 130 | 131 | 1 | 261 | 262 | 0.38 | 0 | 0 | 21 | 21 | 0 | 42 | 42 | 0.00 | 1 | 0 | 109 | 110 | 1 | 219 | 220 | 0.45 | 14 | 0 | 2189 | 2203 | 14 | 4392 | 4406 | 0.32 |
| 3:52223167 | rs35654187 | C>T | c.1149G>A | p.(T383=) | unknown | disrupt | 1 | 0 | 130 | 131 | 1 | 261 | 262 | 0.38 | 0 | 0 | 21 | 21 | 0 | 42 | 42 | 0.00 | 1 | 0 | 109 | 110 | 1 | 219 | 220 | 0.45 | 45 | 0 | 2158 | 2203 | 45 | 4361 | 4406 | 1.02 |
| 3:52222681 | rs352140 | C>T | c.1635G>A | p.(P545=) | unknown | disrupt | 54 | 14 | 63 | 131 | 82 | 180 | 262 | 31.30* | 10 | 2 | 9 | 21 | 14 | 28 | 42 | 33.33* | 44 | 12 | 54 | 110 | 68 | 152 | 220 | 30.91* | 995 | 263 | 945 | 2203 | 1521 | 2885 | 4406 | 34.52* |
| 3:52222605 | rs115440379 | C>T | c.1711G>A | p.(V571M) | probably-damaging:0.992 | disrupt | 1 | 0 | 130 | 131 | 1 | 261 | 262 | 0.38 | 0 | 0 | 21 | 21 | 0 | 42 | 42 | 0.00 | 1 | 0 | 109 | 110 | 1 | 219 | 220 | 0.45 | 17 | 0 | 2186 | 2203 | 17 | 4389 | 4406 | 0.39 |
| 3:52222206 | rs145698725 | G>A | c.2110C>T | p.(R704W) | benign:0.058 | disrupt | 1 | 0 | 130 | 131 | 1 | 261 | 262 | 0.38 | 0 | 0 | 21 | 21 | 0 | 42 | 42 | 0.00 | 1 | 0 | 109 | 110 | 1 | 219 | 220 | 0.45 | 2 | 0 | 2201 | 2203 | 2 | 4404 | 4406 | 0.05 |
| 3:52221826 | rs372418469 | C>G | c.2490G>C | p.(L830=) | unknown | no affect | 1 | 0 | 130 | 131 | 1 | 261 | 262 | 0.38 | 0 | 0 | 21 | 21 | 0 | 42 | 42 | 0.00 | 1 | 0 | 109 | 110 | 1 | 219 | 220 | 0.45 | 2 | 0 | 2200 | 2202 | 2 | 4402 | 4404 | 0.05 |
| 3:52221731 | rs148303873 | C>T | c.2585G>A | p.(G862E) | benign:0.414 | no affect | 2 | 0 | 129 | 131 | 2 | 260 | 262 | 0.76 | 0 | 0 | 21 | 21 | 0 | 42 | 42 | 0.00 | 2 | 0 | 108 | 110 | 2 | 218 | 220 | 0.91 | 20 | 0 | 2183 | 2203 | 20 | 4386 | 4406 | 0.45 |
| 3:52221728 | rs5743845 | C>T | c.2588G>A | p.(R863Q) | benign:0.041 | disrupt | 9 | 1 | 121 | 131 | 11 | 251 | 262 | 4.20 | 1 | 0 | 20 | 21 | 1 | 41 | 42 | 2.38 | 8 | 1 | 101 | 110 | 10 | 210 | 220 | 4.55 | 147 | 4 | 2052 | 2203 | 155 | 4251 | 4406 | 3.52 |
| 3:52221726 | - | C>T | c.2590G>A | p.(D864N) | benign:0.001 | no affect | 1 | 0 | 130 | 131 | 1 | 261 | 262 | 0.38 | 0 | 0 | 21 | 21 | 0 | 42 | 42 | 0.00 | 1 | 0 | 109 | 110 | 1 | 219 | 220 | 0.45 | 0 | 0 | 2203 | 2203 | 0 | 4406 | 4406 | 0.00 |
| 3:52221723 | - | C>T | c.2593G>A | p.(E865K) | benign:0.116 | no affect | 1 | 0 | 130 | 131 | 1 | 261 | 262 | 0.38 | 0 | 0 | 21 | 21 | 0 | 42 | 42 | 0.00 | 1 | 0 | 109 | 110 | 1 | 219 | 220 | 0.45 | 0 | 0 | 2203 | 2203 | 0 | 4406 | 4406 | 0.00 |
| 3:52221697 | - | G>A | c.2619C>T | p.(F873=) | unknown | disrupt | 1 | 0 | 130 | 131 | 1 | 261 | 262 | 0.38 | 0 | 0 | 21 | 21 | 0 | 42 | 42 | 0.00 | 1 | 0 | 109 | 110 | 1 | 219 | 220 | 0.45 | 0 | 0 | 2203 | 2203 | 0 | 4406 | 4406 | 0.00 |
| 3:52221649 | rs149908506 | G>A | c.2667C>T | p.(N889=) | unknown | disrupt | 1 | 0 | 130 | 131 | 1 | 261 | 262 | 0.38 | 0 | 0 | 21 | 21 | 0 | 42 | 42 | 0.00 | 1 | 0 | 109 | 110 | 1 | 219 | 220 | 0.45 | 6 | 0 | 2197 | 2203 | 6 | 4400 | 4406 | 0.14 |
| 3:52221376 | rs445676 | G>A | c.2940C>T | p.(Y980=) | unknown | disrupt | 1 | 0 | 130 | 131 | 1 | 261 | 262 | 0.38 | 0 | 0 | 21 | 21 | 0 | 42 | 42 | 0.00 | 1 | 0 | 109 | 110 | 1 | 219 | 220 | 0.45 | 18 | 0 | 2179 | 2197 | 18 | 4376 | 4394 | 0.41 |

Accession #: NM_017442

* used T allele
